# Tuning Cyclopentadiene Isomerization by Substituent Effects

**DOI:** 10.1007/s00894-026-06871-9

**Published:** 2026-08-05

**Authors:** Agnieszka Łapczuk

**Affiliations:** Cracow University of Technology, Faculty of Chemical Engineering and Technology, 24 Warszawska St., Cracow, PL-31-155 Poland

**Keywords:** 1,5-Hydrogen shift, Sigmatropic rearrangements, Isomerization pathways, Cyclopentadiene, Substituent effects, DFT calculation

## Abstract

**Context:**

The isomerization of substituted cyclopentadienyl systems plays an important role in understanding substituent effects on sigmatropic rearrangements and proton transfer processes. In this work, R- and proton-shifts in substituted cyclopentadienes (MeCp, tBuCp, SiMe_3_Cp, NO_2_Cp, and NH_2_) were systematically investigated. The R-shift proceeds via a single-step pathway, with the nature of the interaction between the migrating group and the ring strongly dependent on the substituent. For MeCp and NH_2_Cp, the migrating group remains covalently bound throughout the process, whereas bulky and electron-withdrawing substituents (tBu, SiMe_3_, NO_2_) lead to polarized transition states with partial charge separation and elongated bonds. Activation barriers are moderate to high, with the lowest values observed for SiMe_3_Cp, and all reactions are exergonic. Proton migration follows a consistent non-classical mechanism involving a transient trisynaptic V(C,C,H) basin and formation of a new C–H bond without full proton dissociation. Substituents primarily influence the extent of electronic reorganization, with NO_2_ inducing increased asynchronicity, while the overall mechanism remains preserved. All processes can be formally classified as [1,5]-sigmatropic rearrangements.

**Methods:**

All calculations were performed using the Gaussian 16 software package. Geometry optimizations and energy calculations were carried out at the ωB97X-D/6-311+G(d,p) level of theory. Solvent effects (toluene, acetone, nitromethane) were included using the PCM model. Transition states were located using the QST2 method and verified by frequency analysis and intrinsic reaction coordinate (IRC) calculations. Topological analyses of the electron localization function (ELF) were performed with TopMod 09 along the IRC, forming the basis for Bond Evolution Theory (BET) analysis. QTAIM analyses were conducted using the Multiwfn program.

**Supplementary information:**

The online version contains supplementary material available at https://doi.org/10.1007/s00894-026-06871-9.

## Introduction

Cyclopentadiene and its analogues, particularly monosubstituted derivatives, play a central role in both organic and organometallic chemistry. These compounds serve as versatile ligands in organometallic complexes, where they can stabilize metal centers and modulate electronic properties [1]. In organic synthesis, cyclopentadiene derivatives are widely employed in cycloaddition reactions, most notably in Diels-Alder transformations, due to their high reactivity and ability to act as electron-rich dienes [2–5]. Their structural diversity, arising from different substituents, allows fine-tuning of regioselectivity and stereoselectivity in these reactions, making them invaluable tools for constructing complex cyclic frameworks. Consequently, understanding the electronic and steric effects of various substituents on cyclopentadiene analogues is essential for rational design and optimization of both organometallic complexes [6] and synthetic cycloaddition processes [7–9].

In the case of monosubstituted cyclopentadiene analogues RCp, equilibrium mixtures of isomers are commonly encountered in practice as a result of the relatively facile isomerization process. These isomers differ in the position of the substituent within the five-membered ring, which leads to variations in electronic structure and, consequently, in chemical reactivity. Previous studies [10] have demonstrated that individual isomers exhibit markedly different reactivities in cycloaddition reactions, thereby influencing both the kinetics and selectivity of these processes [11]. As a result, the isomeric composition of monosubstituted cyclopentadiene systems plays a crucial role in determining their observed reactivity and must be taken into account when interpreting experimental and theoretical results.

Previous studies have addressed the isomerization of cyclopentadiene analogues using both experimental and theoretical approaches [10, 12–15]; however, a comprehensive and systematic comparison of substituent effects—including alkyl groups, their silyl counterparts, and electron-withdrawing substituents—has not yet been reported. Moreover, the reaction mechanism has not been analyzed within the framework of Bonding Evolution Theory (BET) and Quantum Theory of Atoms in Molecules (QTAIM). The aim of the present work is to provide a systematic and comparative investigation of the influence of alkyl, silyl, and electron-withdrawing substituents on the isomerization of cyclopentadienyl systems, to evaluate solvent effects, and to compare the corresponding reaction mechanisms using BET and QTAIM, thereby organizing and rationalizing the existing knowledge in this field.

## Result and discussion

**Scheme 1:**
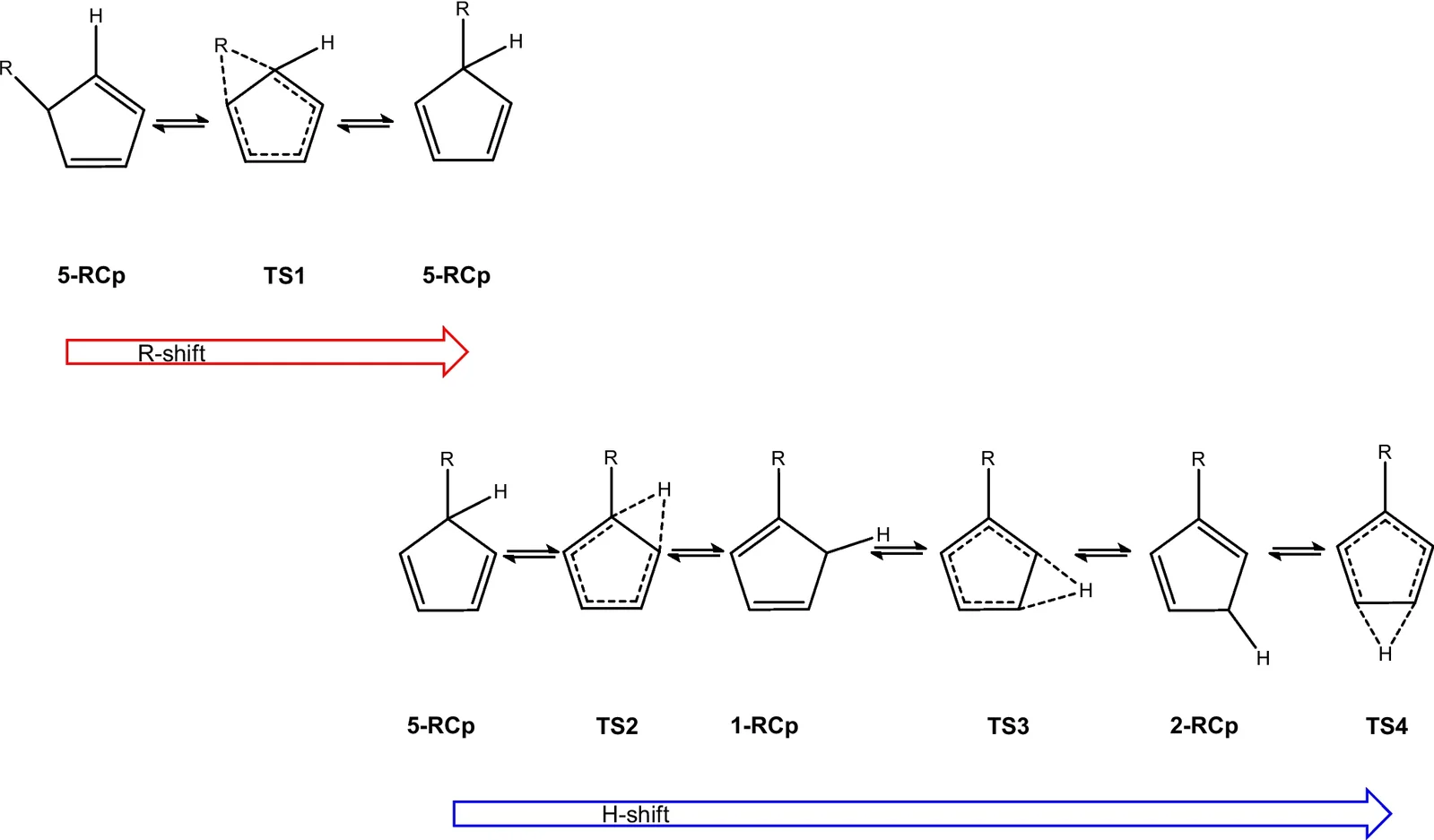
Proposed isomerization mechanism of monosubstituted cyclopentadiene (CpR) via a sigmatropic rearrangement pathway

Although the exact reaction mechanism remains elusive, an extensive computational investigation allowed us to propose a consistent and chemically meaningful mechanistic scenario, shown in Scheme 1.

The mechanism was derived from the identification of all relevant transition states on the potential energy surface, followed by intrinsic reaction coordinate (IRC) calculations to confirm their direct connection to the corresponding minima. The resulting reaction pathway provides a coherent description of the electronic and structural evolution occurring during the transformation.

Isomerization of monosubstituted cyclopentadienes may proceed via proton transfer, corresponding to a 1,5-hydrogen shift along the 5-RCp → TS2 → 1-RCp → TS3 → 2-RCp → TS4 pathway, as well as via migration of the substituent R through an alternative transition state, denoted as TS1. All plausible isomerization pathways were systematically examined and compared in order to determine which RCp isomer is thermodynamically most stable and to assess the extent to which substituent effects govern this stability.

To this end, substituents representing distinct steric and electronic characteristics were selected: a simple alkyl group (Me), a bulky alkyl substituent (tBu), its silyl analogue (SiMe_3_), and, for comparison, a strongly electron-withdrawing substituent (NO_2_) and electron-donating group (NH_2_). This selection enables a comprehensive evaluation of steric and electronic contributions to the isomerization behavior of monosubstituted cyclopentadienes.

### Energy profiles and solvent effects on the isomerization mechanism

Isomerization of cyclopentadiene derivatives RCp may proceed via two distinct mechanistic pathways, involving either proton transfer within the Cp ring or an alternative rearrangement pathway associated with a formal relocation of the substituent R. These pathways do not occur sequentially but instead represent competing routes leading to different local minima on the potential energy surface. Thermodynamic and kinetic parameters were analyzed for five representative substituents, R = tBu, SiMe_3_, Me, NO_2_, and NH_2_. Global energies are referenced to the initial reactant, whereas stepwise barriers are referenced to the preceding intermediate.

The pathway associated with formal substituent relocation is characterized, in the case of MeCp (Table 1), by a high free-energy barrier (ΔG^‡^ ≈ 42–43 kcal/mol) and an entropy change that is close to zero, indicating a highly ordered transition state involving a reorganization of the C–R bond and the π-electron framework of the Cp ring. Owing to its substantial energetic cost, this pathway is expected to be accessible only under high-energy conditions.

**Table 1:** Calculated enthalpy (Δ*H*), Gibbs free energy (Δ*G*), and entropy (Δ*S*) values for stationary points along the isomerization pathways of MeCp in different solvents (toluene, nitromethane, and acetone), obtained at the ωB97X-D/6–311+G(d,p) level of theory using the PCM solvation model. All energies are reported relative to the 5-MeCp isomer within each solvent. Δ*H* and Δ*G* are given in kcal·mol^−1^, and Δ*S* in cal·mol^−1^·K^−1^

	**Toluene**			**Acetone**			**Nitromethane**		
	**Δ*H***	**Δ*G***	**Δ*S***	**Δ*H***	**Δ*G***	**Δ*S***	**Δ*H***	**Δ*G***	**Δ*S***
**5-MeCp → TS1**	42.5	43.1	−2.1	41.8	42.4	−2.1	41.8	42.4	−2.1
**5-MeCp → TS2**	22.3	22.4	−0.5	21.9	22.0	−0.5	21.8	22.0	−0.5
**5-MeCp → 1-MeCp**	−3.3	−3.7	1.1	−3.4	−3.7	1.1	−3.4	−3.7	1.1
**5-MeCp → TS3**	22.9	22.5	1.4	22.6	22.2	1.5	22.6	22.1	1.5
**5-MeCp → 2-MeCp**	−3.5	−3.7	0.9	−3.4	−3.7	0.9	−3.4	−3.7	0.9
**5-MeCp → TS4**	22.1	21.9	0.9	21.8	21.5	0.8	21.8	21.5	0.8

In contrast, the alternative isomerization pathway involves proton transfer between carbon atoms of the Cp ring, with significantly lower activation free energies (ΔG^‡^≈ 21–22 kcal/mol). The corresponding transition states exhibit near-zero entropy changes, consistent with a more localized and less restrictive reorganization of bonding. The nature of these transition states is similar to that previously observed for the isomerization of N-nitropyrazoles [16]. This mechanism enables efficient interconversion between pre-existing RCp isomers without requiring physical migration of the substituent.

Both pathways exhibit only minor sensitivity to solvent polarity, suggesting a predominantly neutral electronic character and the absence of pronounced charge separation in the associated transition states. The observed energetic differences between the two mechanisms are therefore attributed primarily to differences in the extent of structural reorganization within the Cp framework, rather than to solvation effects.

For the SiMe_3_-substituted derivative (Table 2), the pathway associated with formal relocation of the substituent R proceeds with an exceptionally low activation free energy (ΔG^‡^ ≈ 17 kcal/mol), significantly lower than that observed for all other systems investigated. At the same time, this process is characterized by a strongly negative entropy change (ΔS ≈ −7 cal·mol^−1^·K^−1^), indicative of a highly ordered transition state.

**Table 2:** Calculated enthalpy (Δ*H*), Gibbs free energy (Δ*G*), and entropy (Δ*S*) values for stationary points along the isomerization pathways of CpSiMe_3_ in different solvents (toluene, nitromethane, and acetone), obtained at the ωB97X-D/6–311+G(d,p) level of theory using the PCM solvation model. All energies are reported relative to the 5-SiMe_3_Cp isomer within each solvent. Δ*H* and Δ*G* are given in kcal·mol^−1^, and Δ*S* in cal·mol^−1^·K^−1^

	**Toluene**			**Acetone**			**Nitromethane**		
	Δ***H***	Δ***G***	Δ***S***	Δ***H***	**Δ*G***	**Δ*S***	**Δ*H***	**Δ*G***	**Δ*S***
**5-SiMe**_**3**_**Cp → TS1**	15.2	17.2	−6.6	15.1	17.2	−7.3	15.1	17.3	−7.6
**5-SiMe**_**3**_**Cp → TS2**	25.6	26.0	−1.4	25.4	26.0	−2.0	25.4	26.0	−2.0
**5-SiMe**_**3**_**Cp → 1-SiMe**_**3**_**Cp**	2.1	2.2	−0.4	2.3	2.6	−1.0	2.3	2.6	−1.2
**5-SiMe**_**3**_**Cp → TS3**	27.0	27.1	−0.3	26.7	26.8	−0.3	26.7	26.8	−0.2
**5-SiMe**_**3**_**Cp → 2-SiMe**_**3**_**Cp**	3.0	3.3	−0.8	3.2	3.6	−1.6	3.2	3.7	−1.7
**5-SiMe**_**3**_**Cp → TS4**	27.5	29.6	−7.3	27.2	29.6	−8.1	27.2	29.7	−8.3

The unusually low activation barrier reflects the high mobility of the SiMe_3_ group, which can be attributed to the relatively weak C–Si bond and to the ability of silicon to stabilize transient electronic reorganization in the transition state. As a result, substituent relocation represents the most accessible isomerization pathway for the SiMe_3_Cp system.

In contrast, proton-transfer pathways in SiMe_3_Cp require substantially higher activation free energies (ΔG^‡^ ≈ 26–30 kcal/mol). The associated entropy changes are moderately negative, with TS4 exhibiting a particularly large entropic penalty, suggesting a strongly constrained geometry in this transition state.

Both 1-SiMe_3_Cp and 2-SiMe_3_Cp are thermodynamically less stable than the 5-substituted isomer (Δ*G* > 0). Consequently, isomerization of SiMe_3_Cp is thermodynamically disfavored, and any observable rearrangements are governed by kinetic rather than thermodynamic control.

The tert-butyl substituent (Table 3) exhibits the highest activation free energy for substituent relocation within the entire series (ΔG^‡^ ≈ 45–46 kcal/mol). At the same time, the associated entropy change is only moderately negative, indicating that the dominant contribution to the barrier is energetic rather than entropic in origin and is consistent with substantial steric demand.

**Table 3:** Calculated enthalpy (Δ*H*), Gibbs free energy (Δ*G*), and entropy (Δ*S*) values for stationary points along the isomerization pathways of tBuCp in different solvents (toluene, nitromethane, and acetone), obtained at the ωB97X-D/6–311+G(d,p) level of theory using the PCM solvation model. All energies are reported relative to the 5-tBuCp isomer within each solvent. Δ*H* and Δ*G* are given in kcal·mol^−1^, and Δ*S* in cal·mol^−1^·K^−1^

	Toluene			Acetone			Nitromethane		
	Δ***H***	Δ***G***	Δ***S***	Δ***H***	Δ***G***	Δ***S***	Δ***H***	Δ***G***	Δ***S***
**5-tBuCp → TS1**	45.3	46.2	−3.2	44.5	45.4	−2.8	44.5	45.3	−2.8
**5-tBuCp → TS2**	22.5	22.5	0.1	22.1	22.1	0.2	22.1	22.0	0.2
**5-tBuCp → 1-tBuCp**	−2.5	−2.8	1.1	−2.5	−2.8	1.0	−2.5	−2.8	0.9
**5-tBuCp → TS3**	23.5	23.7	−0.5	23.2	23.3	−0.3	23.1	23.2	−0.3
**5-tBuCp → 2-tBuCp**	−2.9	−2.8	−0.3	−2.9	−2.8	−0.2	−2.9	−2.8	−0.2
**5-tBuCp → TS4**	23.1	23.1	0.0	22.7	22.7	0.0	22.7	22.7	0.0

Such a high activation barrier renders tert-butyl relocation unlikely under typical experimental conditions, suggesting that this pathway does not represent a viable isomerization route for tBuCp derivatives.

In contrast, proton-transfer pathways proceed with significantly lower activation free energies (ΔG^‡^≈22–24 kcal/mol) and are therefore kinetically more accessible than substituent relocation. The corresponding entropy changes are close to zero or slightly positive, consistent with localized and relatively unconstrained transition states.

Relocation of the nitro substituent proceeds with an intermediate activation free energy (ΔG^‡^ ≈ 31–36 kcal/mol) (Table 4), clearly lower than that calculated for the tert-butyl derivative, yet higher than for the SiMe_3_-substituted system. A pronounced solvent effect is observed: in polar solvents, the activation barrier is reduced by approximately 5 kcal/mol relative to toluene.

**Table 4:** ωB97X-D/6–311+G(d,p), calculated enthalpy (Δ*H*), Gibbs free energy (Δ*G*), and entropy (Δ*S*) values for stationary points along the isomerization pathways of NO_2_Cp in different solvents (toluene, nitromethane, and acetone). All energies are reported relative to the 5-NO_2_Cp isomer within each solvent. Δ*H* and Δ*G* are given in kcal·mol^−1^, and Δ*S* in cal·mol^−1^·K^−1^

	**Toluene**			**Acetone**			**Nitromethane**		
	**Δ*H***	**Δ*G***	Δ***S***	**Δ*H***	**Δ*G***	**Δ*S***	**Δ*H***	Δ***G***	**Δ*S***
**5-NO**_**2**_**Cp → TS1**	35.1	36.4	−4.2	30.5	31.5	−3.4	30.5	31.5	−3.5
**5-NO**_**2**_**Cp → TS2**	24.0	25.2	−3.9	24.2	25.2	−3.5	24.2	25.2	−3.5
**5-NO**_**2**_**Cp → 1-NO**_**2**_**Cp**	−6.6	−5.8	−2.6	−6.9	−6.2	−2.3	−6.9	−6.2	−2.3
**5-NO**_**2**_**Cp → TS3**	17.1	18.3	−3.9	16.1	17.1	−3.6	16.0	17.1	−3.7
**5-NO**_**2**_**Cp → 2-NO**_**2**_**Cp**	−4.9	−4.3	−2.2	−5.0	−4.5	−1.7	−5.0	−4.5	−1.7
**5-NO**_**2**_**Cp → TS4**	18.8	19.8	−3.3	18.2	19.1	−3.0	18.1	19.0	−3.1

The negative entropy changes associated with this pathway indicate a more ordered transition state, reflecting differences in the electronic structure of the transition state relative to the reactants.

In contrast, proton-transfer pathways proceed with relatively low activation free energies (ΔG^‡^≈ 17–25 kcal/mol), with TS3 and TS4 being significantly lower in energy than TS2.

Both 1-NO_2_Cp and 2-NO_2_Cp are substantially more stable thermodynamically than the 5-substituted isomer (Δ*G* down to −6 kcal/mol). Unlike the MeCp and tBuCp systems, this behavior indicates a genuine thermodynamic preference, which is expected to favor the accumulation of isomers formed via proton-transfer pathways.

Relocation of the amino substituent proceeds with a significantly higher activation free energy (ΔG^‡^ ≈ 50–52 kcal/mol) (Table 5), markedly exceeding those observed for both the nitro- and silyl-substituted systems. In contrast to the NO_2_ case, the solvent effect is relatively modest, with polar solvents lowering the activation barrier by only ~2 kcal/mol compared to toluene. This limited sensitivity suggests a less pronounced charge separation in the transition state associated with NH_2_ migration.

**Table 5:** ωB97X-D/6–311+G(d,p), calculated enthalpy (Δ*H*), Gibbs free energy (Δ*G*), and entropy (Δ*S*) values for stationary points along the isomerization pathways of NH_2_Cp in different solvents (toluene, nitromethane, and acetone). All energies are reported relative to the 5-NH_2_Cp isomer within each solvent. Δ*H* and Δ*G* are given in kcal·mol^−1^, and Δ*S* in cal·mol^−1^·K^−1^

	**Toluene**			**Acetone**			**Nitromethane**		
	**Δ*H***	Δ***G***	**Δ*S***	**Δ*H***	**Δ*G***	**Δ*S***	**Δ*H***	**Δ*G***	**Δ*S***
**5-NH**_**2**_**Cp → TS1**	51.6	52.4	−2.8	49.7	50.5	−2.8	49.5	50.4	−2.8
**5-NHO**_**2**_**Cp → TS2**	31.0	31.2	−0.9	29.9	30.2	−1.1	29.8	30.1	−1.1
**5-NH**_**2**_**Cp → 1-NH**_**2**_**Cp**	−9.7	−9.7	0.1	−10.1	−10.1	0.1	−10.1	−10.2	0.1
**5-NH**_**2**_**Cp → TS3**	29.6	29.6	−0.1	28.9	29.0	−0.3	28.9	29.0	−0.3
**5-NH**_**2**_**Cp → 2-NH**_**2**_**Cp**	−9.5	−9.5	0.0	−9.7	−9.7	−0.1	−9.7	−9.7	0.0
**5-NH**_**2**_**Cp → TS4**	26.6	26.9	−1.1	25.9	26.2	−1.2	25.8	26.2	−1.2

The consistently negative entropy of activation (ΔS ≈ −2.8 cal·mol^−1^·K^−1^) indicates a more ordered transition state, which may reflect a concerted and geometrically constrained rearrangement during amino group transfer. Unlike the NO_2_ system, where electronic delocalization within the substituent may contribute to stabilization, the NH_2_ group appears to undergo a less extensive electronic reorganization, and the energetic cost of migration remains high.

In contrast, proton-transfer pathways are associated with substantially lower activation free energies (ΔG^‡^ ≈ 26–31 kcal/mol), with TS3 and TS4 representing the lowest-energy pathways. These processes exhibit only a weak dependence on solvent polarity and are characterized by near-zero entropy changes (Δ*S* from −1.2 to 0.1 cal·mol^−1^·K^−1^), indicating relatively minor structural reorganization in the transition states.

Both 1-NH_2_Cp and 2-NH_2_Cp are significantly more stable than the parent 5-NH_2_Cp isomer (Δ*G* ≈ −9.5 to −10.1 kcal/mol), with only marginal differences between the two products. This suggests that, despite the high barrier associated with direct amino migration, the overall transformation is thermodynamically favorable. However, in contrast to the NO_2_-substituted system, the large kinetic barrier for NH_2_ transfer is expected to strongly disfavor this pathway, making proton-transfer routes the dominant mechanism under typical conditions.

**Fig. 1:**
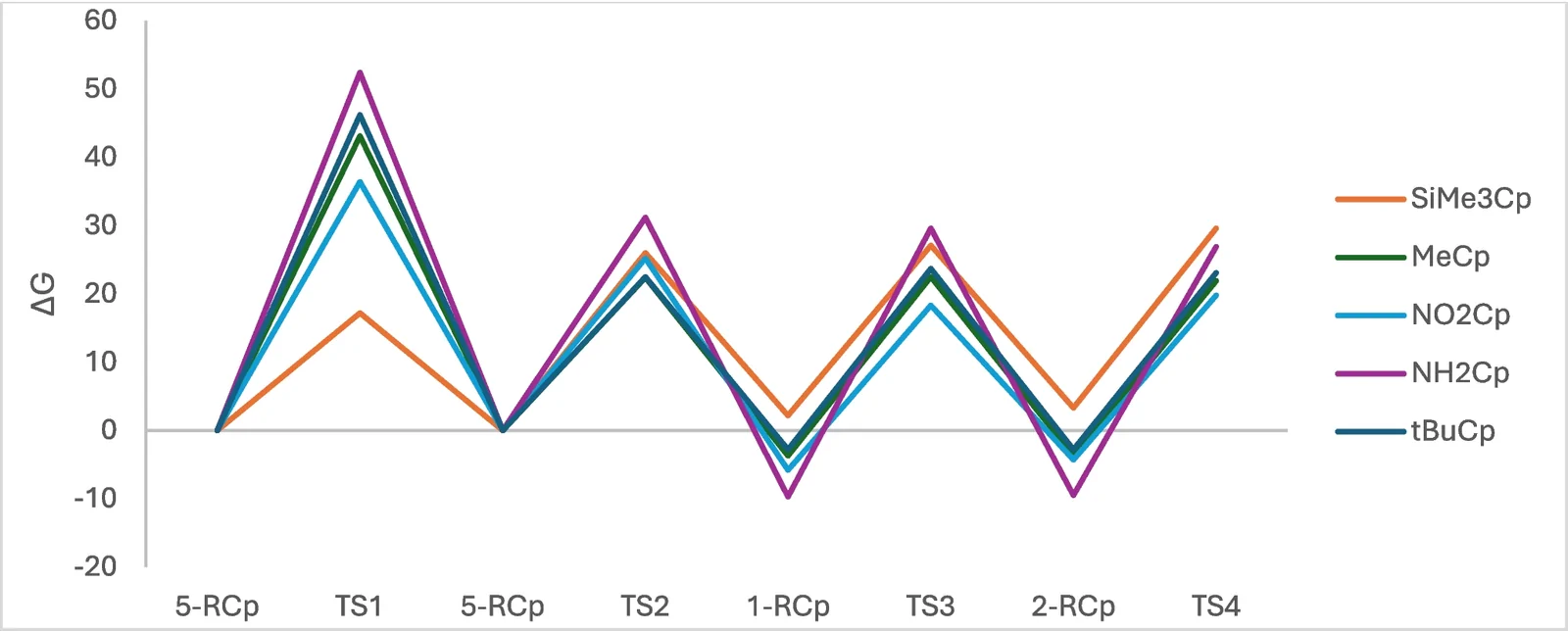
Gibbs free energy profiles (Δ*G*) for the isomerization reaction of substituted cyclopentadienes along the reaction coordinate calculated in toluene

Across the RCp series, R- and H-migration represent competing, non-sequential isomerization mechanisms whose relative importance is dictated by substituent effects (Fig. 1). R-migration is favored for substituents with high migratory aptitude and flexible bonding (SiMe_3_), suppressed by steric bulk (tBu), and moderately accessible for electronically active groups (NO_2_). In the case of the electron-donating NH_2_ group, R-migration remains accessible but is characterized by a more localized and less delocalized bonding rearrangement, reflecting its strong donor character. In contrast, H-migration represents the preferred pathway for all investigated systems in which R-migration is energetically unfavorable, whereas R-migration is favored only in the case of the SiMe_3_ substituent.

**Fig. 2:**
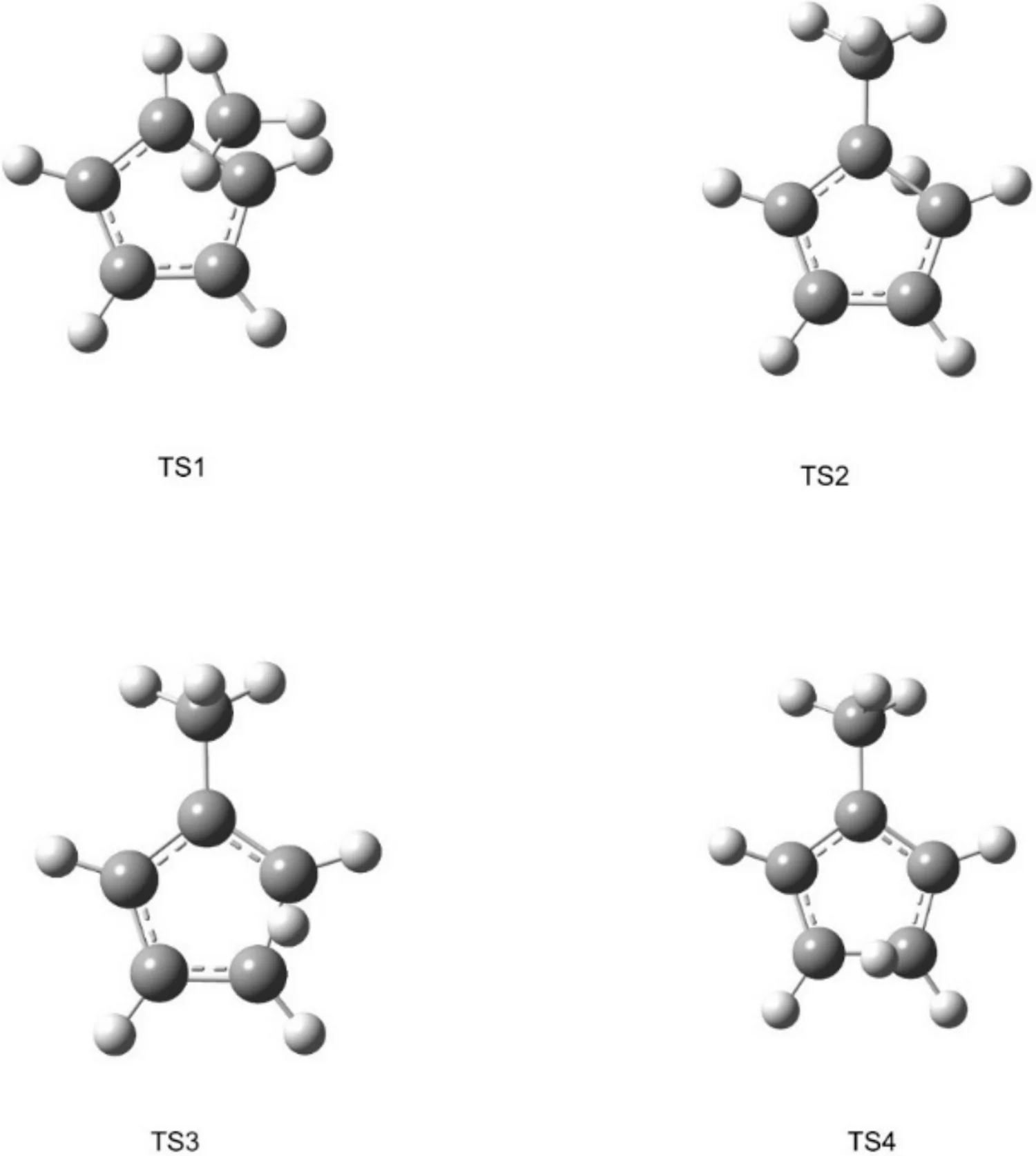
ωB97X-D/6–311+G(d,p): Geometries of the critical structures of transition states of isomerization MeCp

**Fig. 3:**
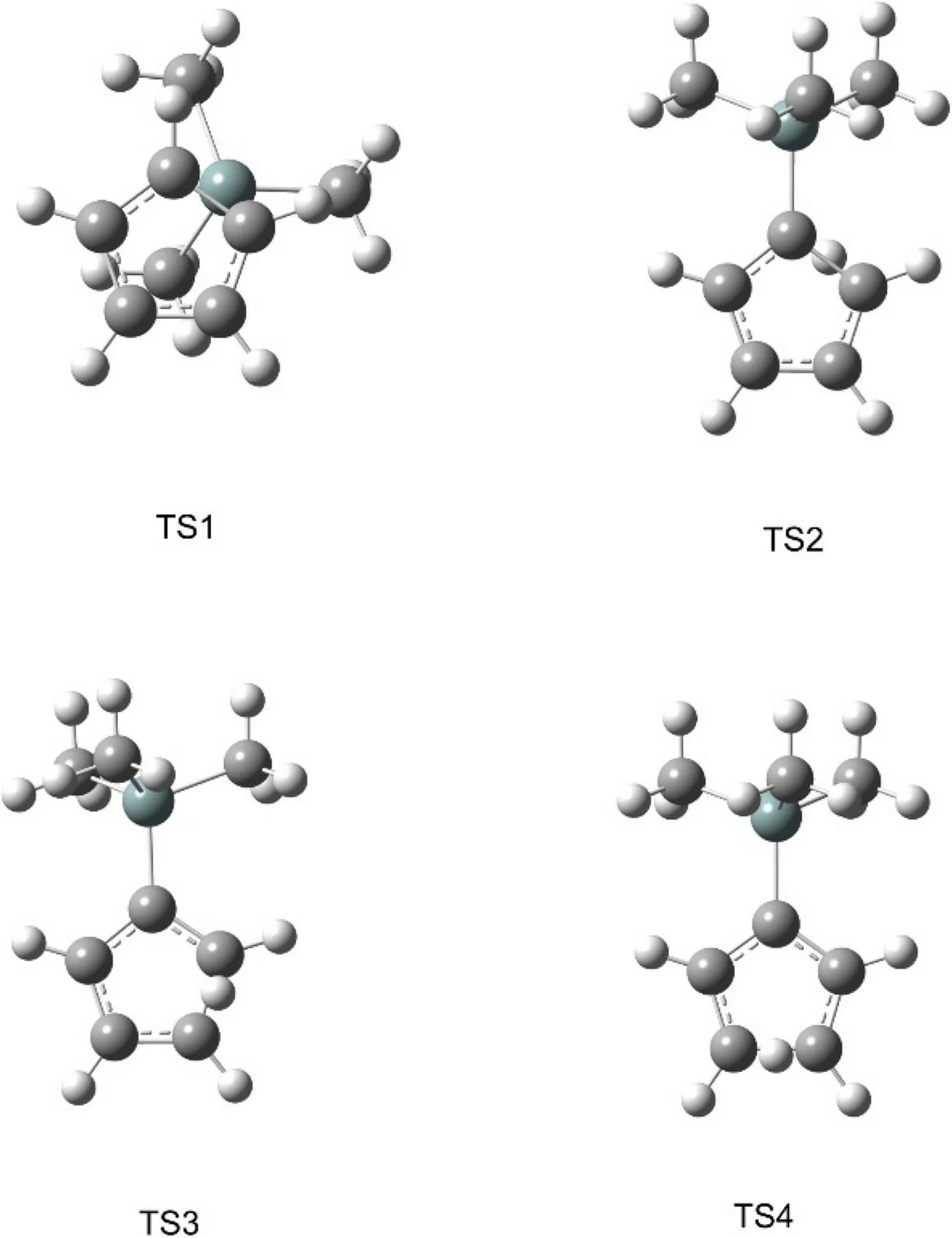
ωB97X-D/6–311+G(d,p): Geometries of the critical structures of transition states of isomerization SiMe_3_Cp

**Fig. 4:**
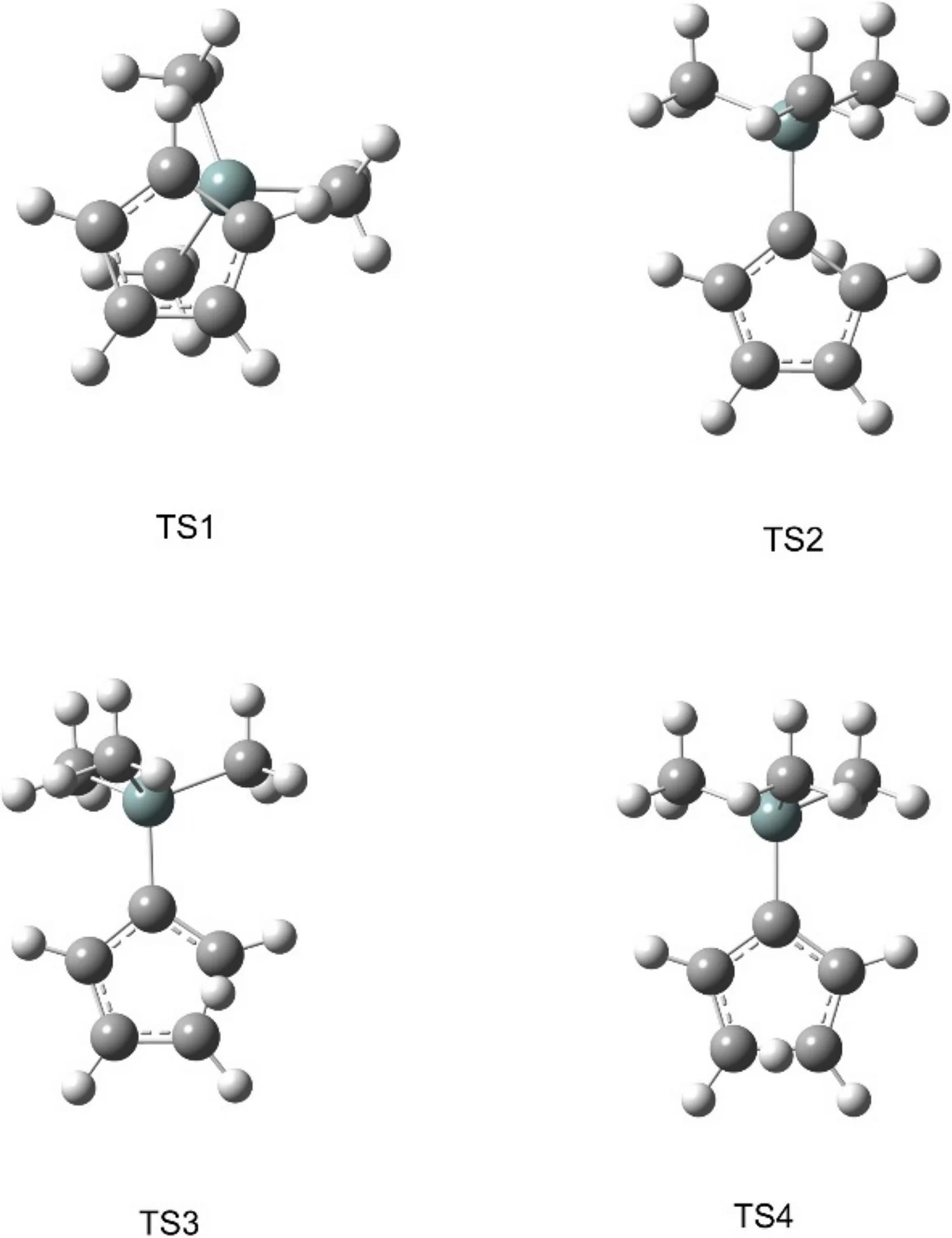
ωB97X-D/6–311+G(d,p): Geometries of the critical structures of transition states of isomerization tBuCp

**Fig. 5:**
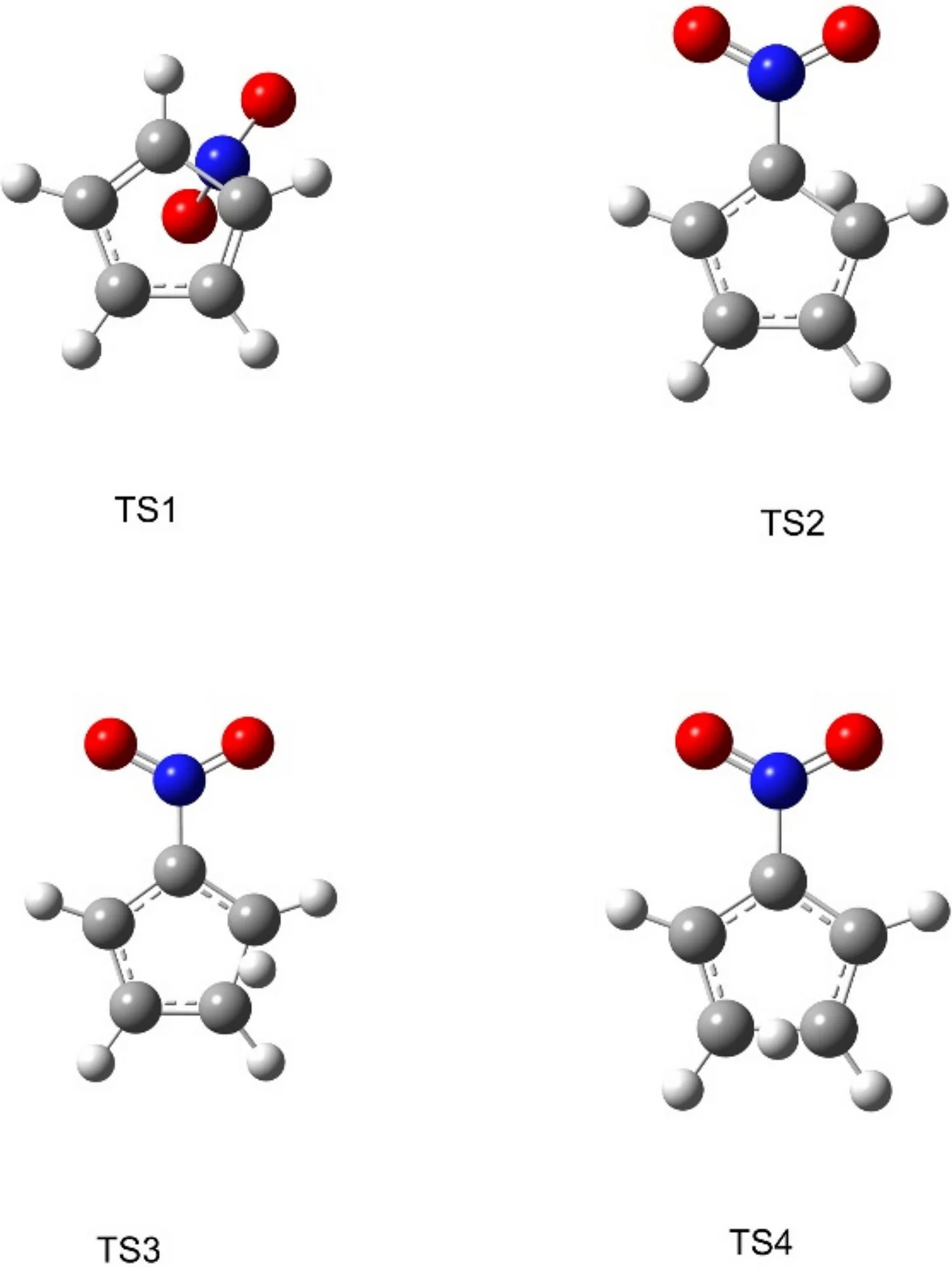
ωB97X-D/6–311+G(d,p): Geometries of the critical structures of transition states of isomerization NO_2_Cp

**Fig. 6:**
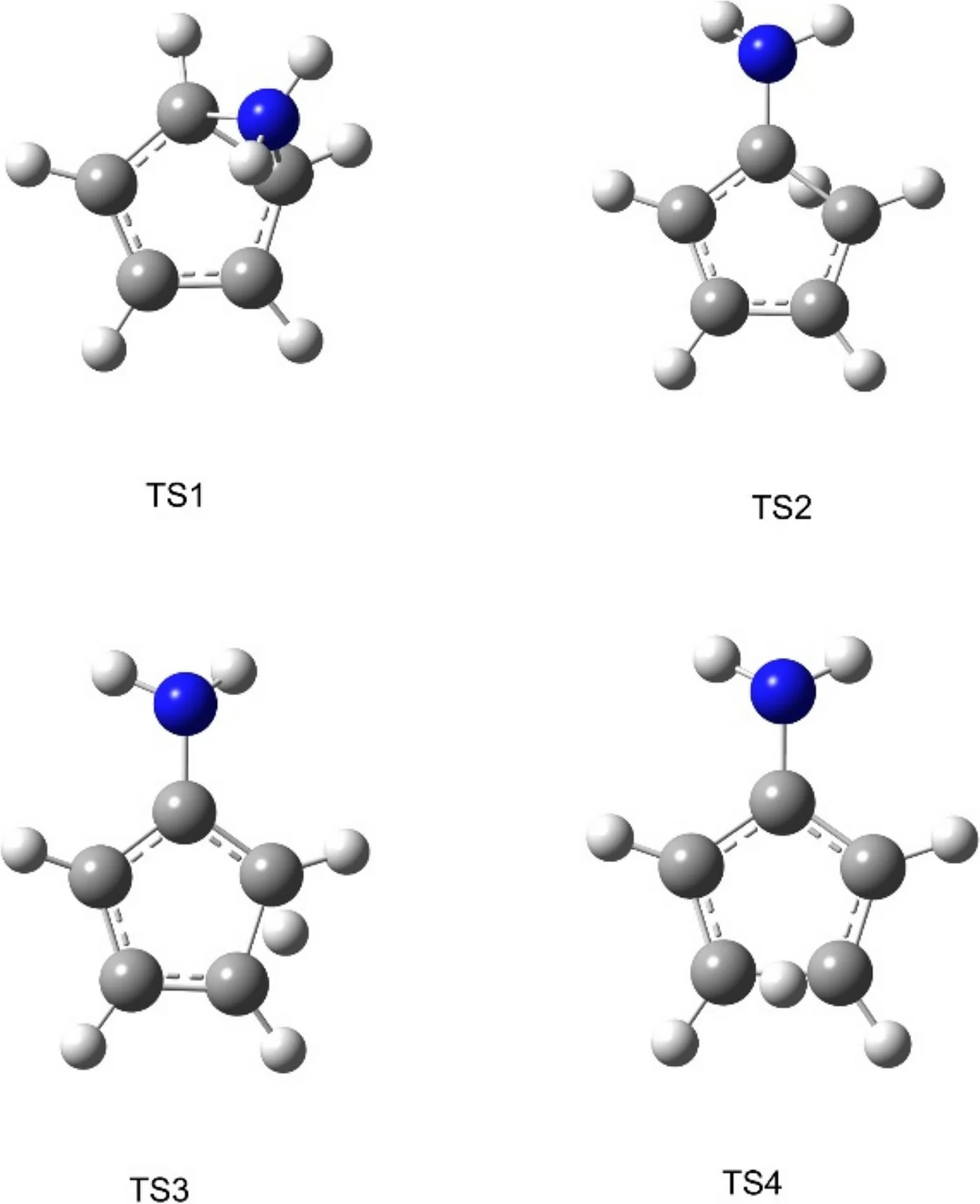
ωB97X-D/6–311+G(d,p): Geometries of the critical structures of transition states of isomerization NH_2_Cp

The weak dependence of most barriers on solvent polarity indicates a predominantly neutral character for both mechanisms, with notable exceptions in systems featuring strong electronic substituents. Overall, the observed trends underscore the delicate balance between steric, electronic, and entropic factors in governing isomerization pathways in substituted cyclopentadienes. Geometries of the critical structures of transition states of isomerization RCp are shown in Figs. 2, 3, 4, 5, and 6.

### Mechanism of the isomerization reaction elucidated by Bonding Evolution Theory (BET)

Bonding Evolution Theory (BET), based on the topology of the electron localization function (ELF), provides a framework for analyzing changes in chemical bonding along the reaction coordinate. In this approach, the electron density is partitioned into basins associated with attractors (local maxima of ELF), which can be interpreted in terms of chemical entities such as bonds or lone pairs.

To gain detailed insight into the reaction mechanism and the evolution of chemical bonds during the isomerization process, we performed an analysis based on Bond Evolution Theory (BET) [17]. This approach enables a precise characterization of bond formation and cleavage along the reaction pathway, providing a clear picture of electron density redistribution during the transformation. By applying BET, we aimed to identify the key stages of the reaction, elucidate the sequence of bond-making and bond-breaking events, and assess how systems bearing different substituents may behave.

#### Migration of substituent groups (R-shift) along the reaction path

The migration of the methyl group in MeCp from C5 to C1 was analyzed using ELF within BET. The IRC structures reveal a sequence of topological changes associated with the breaking of the C5-C6 bond and the formation of a new C1-C6 bond. Disynaptic basins are associated with two atomic centers and correspond to conventional two-center bonds. In contrast, trisynaptic basins involve three atomic centers and typically arise in situations where electron density is shared among more than two atoms, such as in multicenter interactions or during bond reorganization processes. At the beginning of the reaction path, the methyl group is bonded to C5, as evidenced by the disynaptic basin V(C5,C6) with a population of 1.75 e. As the reaction progresses, the population of this basin decreases to 1.28 e, indicating progressive weakening of the C5-C6 bond. A key topological event occurs with the appearance of a trisynaptic basin V(C1,C5,C6) integrating 1.01 e, which indicates that the migrating methyl carbon simultaneously interacts with both carbon centers. This shared-electron configuration characterizes the transition region of the migration process. Further along the reaction coordinate, the trisynaptic basin disappears and a new disynaptic basin V(C1,C6) emerges, whose population increases from 0.97 to 1.75 e, confirming the formation of the new C1-C6 σ bond. Simultaneously, significant redistribution of electron density occurs within the carbon framework, particularly in the V(C2,C3) and V(C4,C5) basins, reflecting transient delocalization followed by re-localization of electron density during the structural rearrangement. The appearance of trisynaptic basins is associated with stages of the reaction where electron density becomes delocalized over multiple atomic centers, particularly in transition states or during bond rearrangement. In contrast, in well-defined bonding situations, disynaptic basins dominate, reflecting localized two-center interactions.

**Scheme 2:**
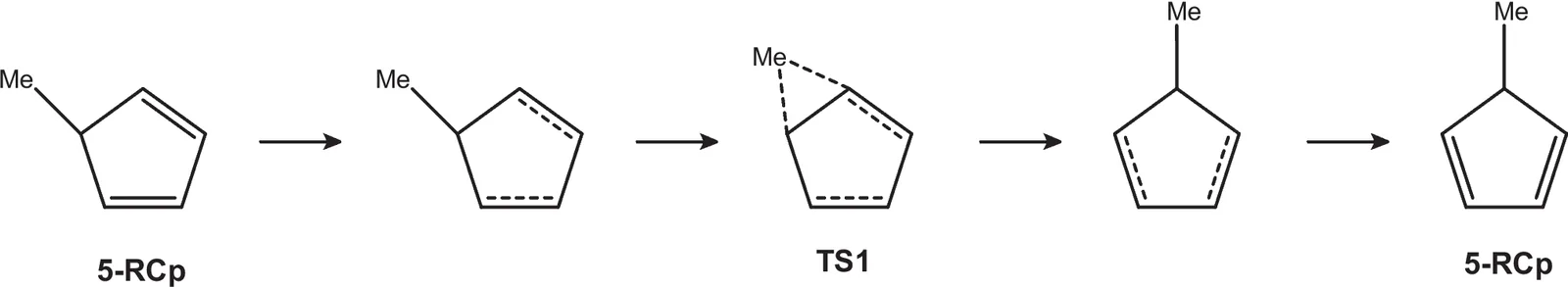
Simplified representation of the molecular mechanism of the isomerization reaction of MeCp using ELF-based Lewis structures

Overall, the ELF analysis indicates that the methyl migration proceeds through a transition region characterized by a transient trisynaptic basin rather than a classical stepwise bond-breaking and bond-forming process (Scheme 2, Table 6).

**Table 6:** BET analysis of the methyl migration along the intrinsic reaction coordinate (IRC) pathway. The BET phases were defined based on the changes in electron populations and molecular topology observed along the reaction coordinate. Distances are given in angstroms (Å) and electron populations in average number of electrons (e)

Phase	1	2	3	4	5	
d1(C5-C6)	1.545	1.767	1.893	1.954	2.070	2.450
d2(C1-C6)	2.451	2.058	1.941	1.881	1.755	1.545
V(C2,C3)	2.01	2.22	2.58	2.96	1.61	1.65
V′(C2,C3)					1.63	1.72
V(C1,C5)	2.02	2.09	2.11	2.16	2.08	2.02
V(C5,C6)	1.75	1.28				
V(C1,C5,C6)			1.01			
V(C1,C6)				0.97	1.32	1.75
V(C3,C4)	1.72	3.25	3.01	2.7	2.37	2.2
V′(C3,C4)	1.65					
V(C4,C5)	2.2	2.4	2.75	3.05	1.66	1.72
V′(C4,C5)					1.61	1.65
V(C1,C2)	1.65	3.21	2.91	2.52	2.21	2.01
V′(C1,C2)	1.72					

**Scheme 3:**
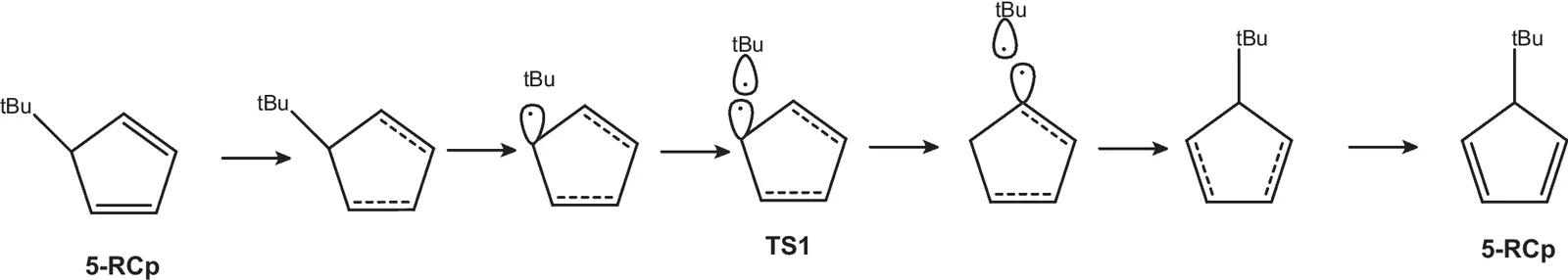
Simplified representation of the molecular mechanism of the isomerization reaction of tBuCp using ELF-based Lewis structures

The tert-butyl substituted system (Table 7, Scheme 3) exhibits a bonding evolution consistent with the migration of the substituent from C5 to C1 along the reaction path. At the beginning of the process (phase 1), the disynaptic basin V(C5,C6) integrates about 1.81 e, corresponding to the C5-C6 σ bond. As the reaction proceeds, this basin progressively depopulates and disappears in phase 4, indicating cleavage of the C5-C6 bond. The bond breaking is accompanied by the appearance of monosynaptic basins V(C5) and V(C6), as well as a small basin V(C1), which reflect the transient localization of electron density during the rearrangement. In the later stages of the reaction coordinate, a new disynaptic basin V(C1,C6) emerges and gradually increases its population from 0.58 to 1.80 e, indicating the formation of the new C1-C6 bond.

**Table 7:** BET analysis of the tert-butyl migration along the intrinsic reaction coordinate (IRC) pathway. The BET phases were defined based on the changes in electron populations and molecular topology observed along the reaction coordinate. Distances are given in angstroms (Å) and electron populations in average number of electrons (e)

Phase	1	2	3	4	5	6	7	8	9	10	
d1(C5-C6)	1.591	1.893	1.927	2.058	2.088	2.155	2.179	2.201	2.270	2.277	2.531
d2(C1-C6)	2.563	2.285	2.270	2.197	2.174	2.106	2.077	2.045	1.909	1.892	1.572
V(C5,C6)	1.81	1.29	1.22	0.59							
V(C5)				0.3	0.21						
V(C6)					0.57	0.56					
V(C1)						0.17	0.26				
V(C1,C6)							0.58	0.93	1.24	1.28	1.8
V(C4,C5)	2.01	2.23	2.27	2.53	2.68	3.03	3.07	3.12	1.59	1.65	1.65
V′(C4,C5)									1.65	1.59	1.71
V(C1,C5)	2.03	2.13	2.16	2.23	2.25	2.25	2.24	2.22	2.14	2.14	2.05
V(C3,C4)	1.66	3.23	3.21	3.04	2.94	2.67	2.6	2.54	2.36	2.35	2.2
V′(C3,C4)	1.71										
V(C2,C3)	2.19	2.33	2.37	2.55	2.61	2.86	2.99	3.06	3.21	1.57	1.7
V′(C2,C3)										1.66	1.65
V(C1,C2)	1.66	1.66	3.22	3.11	3.05	2.76	2.59	2.49	2.25	2.24	2.02
V′(C1,C2)	1.72	1.59

Overall, the BET sequence supports a stepwise redistribution of electron density in which the C5-C6 bond is first weakened and broken, followed by the gradual formation of the C1-C6 bond, consistent with the proposed sigmatropic migration pathway.

**Scheme 4:**
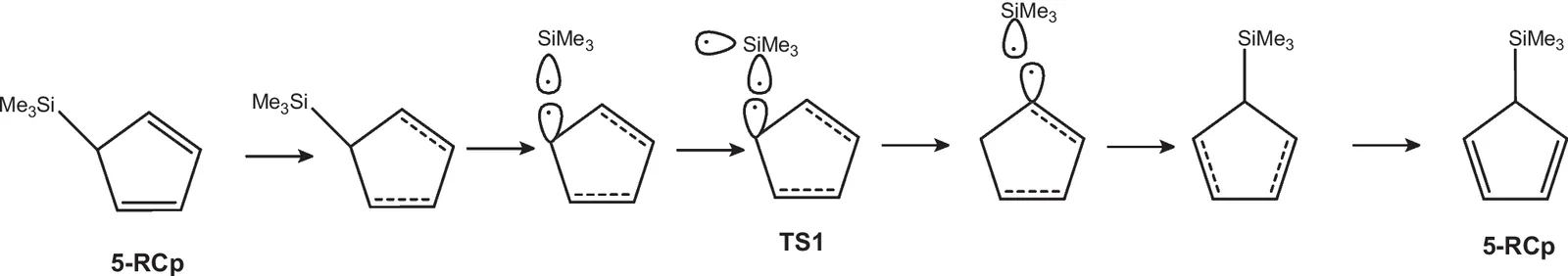
Simplified representation of the molecular mechanism of the isomerization reaction of SiMe_3_Cpusing ELF-based Lewis structures

In the SiMe_3_-substituted cyclopentadiene (Table 8, Scheme 4), the bonding changes are consistent with migration of the silyl group along the reaction coordinate. At the beginning of the process (phase 1), the disynaptic basin V(C4,Si) integrates about 1.81 e, corresponding to the C4-Si bond. As the reaction progresses, the population of this basin decreases and the basin disappears in phase 3, indicating the cleavage of the C4-Si bond.

**Table 8:** BET analysis of the trimetylsillyl migration along the intrinsic reaction coordinate (IRC) pathway. The BET phases were defined based on the changes in electron populations and molecular topology observed along the reaction coordinate. Distances are given in angstroms (Å) and electron populations in average number of electrons (e)

Phase	1	2	3	4	5	6	7	8	9	10	11	12	
d1(Si-C5)	1.933	1.960	1.966	2.058	2.122	2.205	2.216	2.260	2.413	2.527	2.536	2.566	2.747
d2(Si-C1)	2.747	2.556	2.526	2.326	2.248	2.163	2.153	2.112	2.006	1.966	1.964	1.958	1.933
V(C4,Si)	1.81	1.71	1.7										
V(C4)				1.44	1.18	0.65	0.58						
V(Si)				0.76	0.25	0.62	0.63	0.26	0.32				
V′(Si)					0.19	0.19							
V(C1)						0.91	0.98	1.23					
V(C1,Si)									1.59	1.7	1.7	1.72	1.81
V(C1,C2)	1.65	3.23	3.22	3.16	3.24	2.58	2.55	2.42	2.19	2.12	2.11	2.1	2.05
V′(C1,C2)	1.66												
V(C1,C4)	2.06	2.1	2.09	2.14	2.12	2.11	2.12	2.12	2.12	2.09	2.08	2.08	2.06
V(C2,C3)	2.29	2.35	2.37	2.53	2.67	2.86	2.89	2.99	3.16	3.22	1.59	1.63	1.68
V′(C2,C3)											1.65	1.61	1.61
V(C3,C5)	1.68	1.62	3.22	3.1	2.97	2.78	2.74	2.64	2.44	2.37	2.36	2.35	2.3
V′(C3,C5)	1.61	1.62											
V(C4,C5)	2.05	2.11	2.12	2.29	2.45	2.73	2.78	3.23	3.18	3.22	3.23	1.61	1.65
V′(C4,C5)												1.64	1.66

This event is accompanied by the appearance of monosynaptic basins V(C4) and V(Si), as well as a secondary basin V′(Si), reflecting the temporary localization and redistribution of electron density around the silicon atom. Additional monosynaptic density appears at C1 (V(C1)), indicating the development of a reactive center at this carbon atom. In the later stages of the reaction path, a new disynaptic basin V(C1,Si) emerges and progressively increases its population from about 1.59 to 1.81 e, confirming the formation of the new C1-Si bond.

Overall, the BET sequence reveals a progressive electron density reorganization in which the initial C4-Si bond breaks and a new C1-Si bond is formed, while the presence of multiple monosynaptic basins at silicon reflects the high polarizability of the silyl substituent during the rearrangement.

**Scheme 5:**
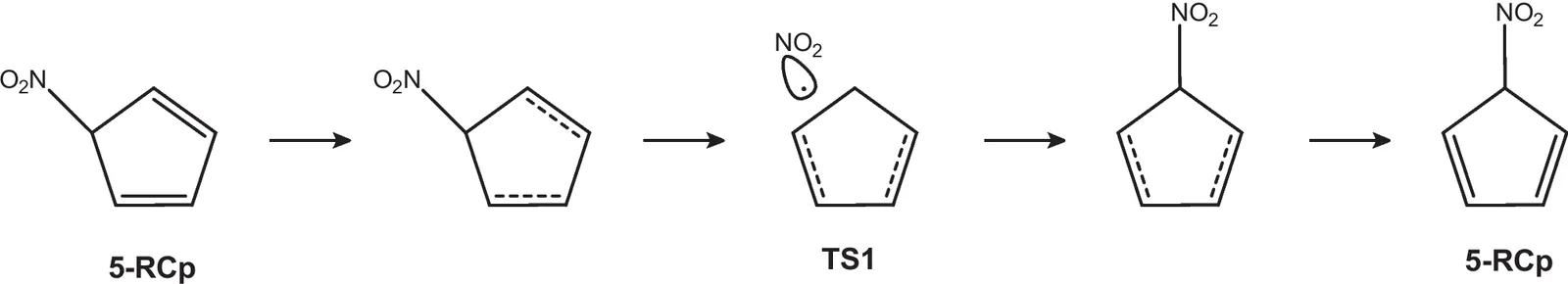
Simplified representation of the molecular mechanism of the isomerization reaction of NO_2_Cp using ELF-based Lewis structures

The BET analysis of the NO_2_-substituted cyclopentadiene (Table 9, Scheme 5) indicates a redistribution of electron density associated with the migration of the nitro group along the reaction coordinate. In the initial structure, the disynaptic basin V(C5,N) integrates about 2.37 e, corresponding to the C5-N bond. As the reaction proceeds, this basin disappears and a monosynaptic basin V(N) emerges in phase 2 with a population of about 2.22 e, indicating the temporary localization of electron density at the nitrogen atom after cleavage of the C5-N bond. In the later stages of the reaction path, a new disynaptic basin V(N,C1) appears with a population of about 2.37–2.39 e, confirming the formation of the new C1-N bond.

**Table 9:** BET analysis of the nitro group migration along the intrinsic reaction coordinate (IRC) pathway. The BET phases were defined based on the changes in electron populations and molecular topology observed along the reaction coordinate. Distances are given in angstroms (Å) and electron populations in average number of electrons (e)

Phase	1	2	3	4	
d1(C5-N)	1.912	1.971	2.126	2.156	2.169
d2(C1-N)	2.156	2.110	1.951	1.912	1.891
V(C1,C2)	3.1	2.95	2.39	2.33	2.31
V(C4,C5)	2.33	2.43	3.02	3.1	3.13
V(C2,C3)	2.43	2.71	1.62	1.64	1.65
V′(C2,C3)			1.57	1.6	1.61
V(C5,N)	2.37				
V(N)		2.22	2.22		
V(C1,N)				2.37	2.39
V(C1,C5)	2.1	2.11	2.1	2.1	2.08
V(C3,C4)	1.64	3.15	2.65	2.43	2.39
V′(C3,C4)	1.6

Overall, the BET sequence reveals a bond reorganization in which the initial C3-N bond is broken and a new C5-N bond is formed through a transient stage characterized by a monosynaptic basin at the nitrogen atom.

**Scheme 6:**
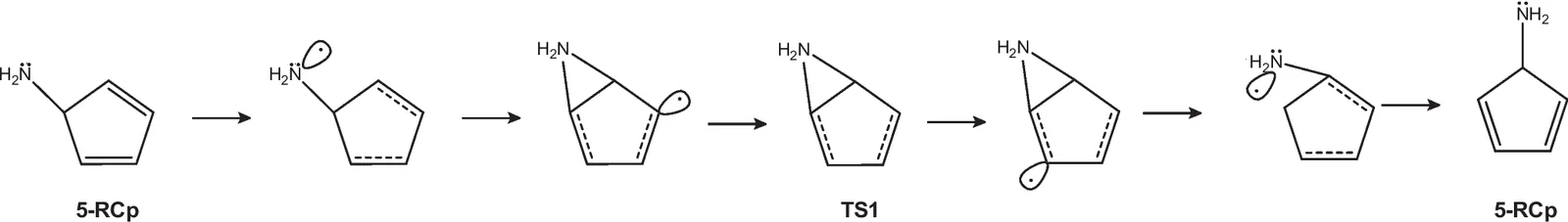
Simplified representation of the molecular mechanism of the isomerization reaction of NH_2_Cp using ELF-based Lewis structures

The NH_2_-substituted system (Table 10, Scheme 6) exhibits a bonding evolution consistent with the migration of the amino group from C5 to C1 along the reaction coordinate. At the beginning of the process (phase 1), the disynaptic basin V(N,C5) integrates about 1.62 e, corresponding to the C5-N bond. As the reaction progresses, the population of this basin gradually decreases and the basin disappears around phase 9, indicating cleavage of the C5-N bond. This bond-breaking process is accompanied by the disappearance of the monosynaptic basin V(N). At the stage where both C1–N and C5–N interactions are established, the monosynaptic basin on the nitrogen atom vanishes completely. It is subsequently restored upon cleavage of the C5–N bond.

**Table 10:** BET analysis of the amino group migration along the intrinsic reaction coordinate (IRC) pathway. The BET phases were defined based on the changes in electron populations and molecular topology observed along the reaction coordinate. Distances are given in angstroms (Å) and electron populations in average number of electrons (e)

Phases	1	2	3	4	5	6	7	8	9	10	11	12	13	
d1(C5-N)	1.468	1.497	1.499	1.513	1.532	1.536	1.576	1.61	1.729	1.747	1.873	1.994	2.026	2.445
d2(C1-N)	2.44	2.01	1.977	1.856	1.729	1.711	1.596	1.567	1.532	1.529	1.511	1.498	1.496	1.467
V(C1,N)					1.65	1.66	1.69	1.74	1.8	1.81	1.49	1.51	1.52	1.62
V(C5, N)	1.62	1.52	1.51	1.84	1.80	1.80	1.74	1.69	1.66					
V(N)	2.14	1.85	1.81	1.68						1.66	1.71	1.83	1.87	2.15
V′(N)		0.25	0.28								0.35	0.27		
V(C2)						0.38								
V(C4)								0.36						
V(C2,C3)	2.16	2.22	2.24	2.33	2.66	2.70	3.35	3.34	3.39	3.39	1.71	1.74	1.74	1.74
V′(C2,C3)											1.68	1.66	1.66	1.66
V(C3,C4)	1.74	1.66	1.66	3.39	3.39	3.38	3.32	2.96	2.66	2.61	2.32	2.23	2.21	2.16
V′(C3,C4)	1.66	1.74	1.74											
V(C1,C2)	1.74	1.76	3.34	3.3	3.01	2.58	2.33	2.27	2.17	2.16	2.1	2.06	2.05	2.04
V′(C1,C2)	1.65	1.60												
V(C1,C5)	2.01	1.99	1.99	1.99	1.94	1.93	1.89	1.89	1.94	1.95	1.98	1.99	2.00	2.01
V(N,H1)	1.98	2.00	2.00	1.99	2.00	2.01	2.02	2.02	2.00	2.00	1.99	2.00	2.21	1.98
V(N,H2)	1.98	2.01	2.00	1.99	2.00	2.00	2.02	2.02	2.00	2.00	1.98	2.00	2.03	1.98
V(C4,C5)	2.04	2.06	2.06	2.11	2.17	2.18	2.29	2.35	3.00	3.04	3.31	1.75	1.76	1.74
V′(C4,C5)												1.61	1.61	1.65

In the intermediate region of the reaction path, the appearance of monosynaptic basins at carbon atoms (V(C2), V(C4)) indicates the development of transient reactive centers within the ring, associated with the reorganization of the π system. In the later stages of the process, a new disynaptic basin V(C1,N) emerges (phase 4) and progressively increases its population from about 1.65 to 1.81 e, confirming the formation of the new C1-N bond. The simultaneous shortening of the C1-N distance (from 2.44 to 1.47 Å) and elongation of the C5-N distance (from 1.47 to 2.45 Å) further support the migration process.

Significant redistribution of electron density is also observed within the carbon framework, particularly in the V(C2,C3) and V(C4,C5) basins, reflecting transient delocalization followed by re-localization of electron density during the structural rearrangement.

Overall, the sequence of topological changes indicates that the NH_2_ migration proceeds through a stepwise reorganization of electron density involving cleavage of the C5-N bond, transient localization of electron density at the nitrogen atom, and subsequent formation of the C1-N bond, without the formation of a pronounced trisynaptic basin.

Overall, the analysis reveals that the migration mechanism strongly depends on the electronic nature of the substituent, leading to distinct patterns of electron density reorganization along the reaction coordinate. For methyl substituent (Me), the migration proceeds through a transition region characterized by the formation of a transient trisynaptic basin, indicating partial delocalization of electron density between three atomic centers. This behavior reflects a non-classical, concerted bond reorganization mechanism.

In contrast, the SiMe_3_ substituent exhibits a more polarized behavior, with the migration involving the formation of multiple monosynaptic basins at the silicon center. This reflects the high polarizability of silicon and its ability to accommodate transient electron density, leading to a more stepwise redistribution process. A similar, though less pronounced, behavior is observed for the tBu substituent, which also shows partial localization of electron density during the migration. This effect may result from a combination of weak electron donation through hyperconjugation and steric influences associated with the bulky tert-butyl group, both of which can affect the redistribution of electron density along the reaction coordinate.

For strongly electron-withdrawing substituents such as NO_2_, the mechanism is dominated by the formation of a monosynaptic basin at the heteroatom after cleavage of the C–N bond, indicating significant localization of electron density on the substituent. This suggests a more ionic character of the intermediate stages of the migration.

In the case of the electron-donating NH_2_ group, the migration proceeds through a stepwise reorganization of electron density without the formation of a pronounced trisynaptic basin. Instead, the process involves the simultaneous formation of two C–N bonds, accompanied by the depletion of electron density from the nitrogen atom. This behavior highlights the strong electron-donating character of NH_2_, which stabilizes localized electron density rather than promoting multicenter delocalization. Unlike the tBu substituent, where both hyperconjugative electron donation and steric bulk contribute to the observed behavior, the effect of NH₂ is primarily attributed to its strong mesomeric electron-donating character, with negligible steric and hyperconjugative contributions.

Taken together, these results demonstrate that the substituent effects are governed by a balance between electron donation, electron withdrawal, and polarizability, which control whether the migration proceeds via a delocalized trisynaptic transition region or through a more localized, stepwise mechanism.

#### Migration of proton (H-shift) along the reaction path

##### Proton migration from C1 to C2 in substituted cyclopentadienes

The Bonding Evolution Theory (BET) analysis reveals that the proton transfer between C1 and C2 proceeds through a qualitatively similar topological sequence for all four cyclopentadiene derivatives; however, distinct quantitative and mechanistic differences emerge depending on the nature of the substituent.

For all systems, the reaction is initiated by the progressive weakening of the original C1-H bond and the concomitant strengthening of the C2-H interaction, as reflected by the monotonic increase of the d2(C2-H) distance and the disappearance of the disynaptic basin V(C1,H). In each case, the proton migration proceeds through the formation of a trisynaptic basin V(C1,C2,H), indicating a non-classical, electron-shared hydrogen transfer mechanism rather than a stepwise heterolytic or homolytic process.

**Scheme 7:**
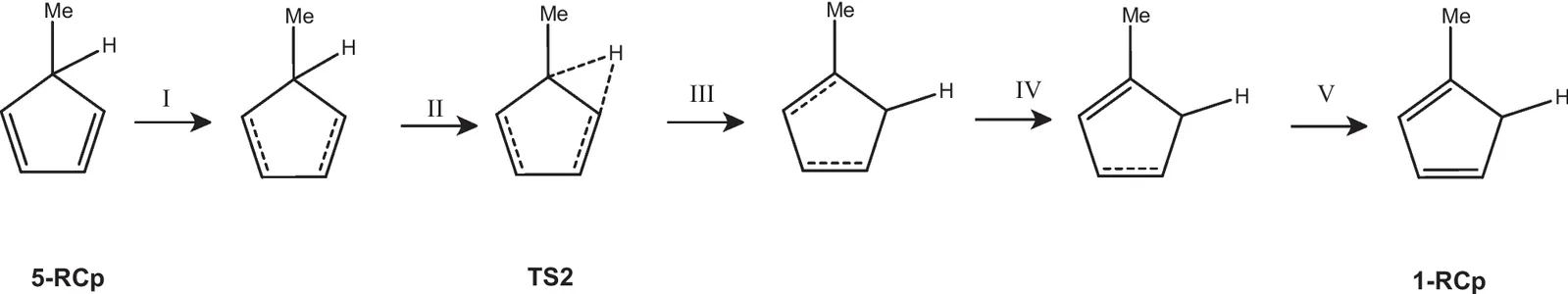
Simplified representation of the molecular mechanism of the isomerization reaction MeCp using ELF-based Lewis structures

**Scheme 8:**
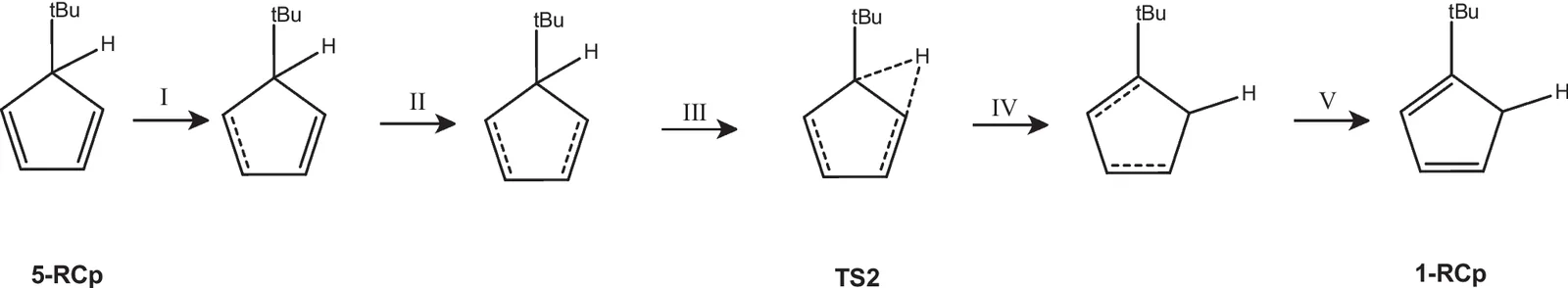
Simplified representation of the molecular mechanism of the isomerization reaction tBuCp using ELF-based Lewis structures

Despite this common qualitative pattern, clear substituent-dependent differences are observed in the population of the trisynaptic basin and in the accompanying reorganization of the carbon-carbon bonding framework. For the Me and tBu substituted systems (Tables 11 and 12, Schemes 7 and 8), the population of the V(C1,C2,H) basin stabilizes at approximately 1.68 e, and the evolution of the neighboring C–C disynaptic basins remains highly symmetric. The maximum populations of the reorganized C–C basins (V(C3,C4), V(C4,C5), or V(C1,C5), depending on the stage) reach values close to 3.21 e, indicating efficient delocalization within the π-system and a relatively synchronous proton shift.

**Table 11:** BET analysis of the [1, 5]-sigmatropic hydrogen shift between C1 and C2 in MeCp along the intrinsic reaction coordinate (IRC) pathway. The BET phases were defined based on the changes in electron populations and molecular topology observed along the reaction coordinate. Distances are given in angstroms (Å) and electron populations in average number of electrons (e)

Phase	1	2	3	4	5	6	
d1(C1-H)	1.125	1.156	1.223	1.504	1.689	1.713	2.141
d2(C2-H)	2.142	1.704	1.472	1.208	1.156	1.153	1.123
V(C2,C3)	1.66	3.25	3.08	2.3	2.15	2.14	1.99
V′(C2,C3)	1.7						
V(C1,C2)	2.01	2.03	1.99	1.99	2.01	2.01	2.01
V(C1,H)	1.98	1.75					
V(C1,C2,H)			1.68				
V(C2,H)				1.66	1.73	1.74	1.94
V(C3,C4)	2.21	2.39	2.58	3.15	3.25	1.63	1.7
V′(C3,C4)						1.64	1.68
V(C4,C5)	1.7	3.24	3.12	2.54	2.39	2.37	2.21
V′(C4,C5)	1.66						
V(C1,C5)	2.01	2.18	2.37	3.21	1.74	1.67	1.73
V′(C1,C5)					1.59	1.67	1.71

**Table 12:** BET analysis of the [1, 5]-sigmatropic hydrogen shift between C1 and C2 in tBuCp along the intrinsic reaction coordinate (IRC) pathway. The BET phases were defined based on the changes in electron populations and molecular topology observed along the reaction coordinate. Distances are given in angstroms (Å) and electron populations in average number of electrons (e)

Phase	1	2	3	4	5	6	
d1(C1-H)	1.129	1.157	1.161	1.228	1.511	1.719	2.168
d2(C2-H)	2.107	1.698	1.675	1.466	1.206	1.153	1.120
V(C2,C3)	1.66	1.66	3.23	3.07	2.3	2.14	2.0
V′(C2,C3)	1.69	1.59					
V(C1,C2)	2.06	2.04	2.04	1.98	1.99	2.01	2.02
V(C1,H)	1.94	1.75	1.74				
V(C1,C2,H)				1.68			
V(C2,H)					1.66	1.75	1.96
V(C3,C4)	2.22	2.38	2.4	2.59	3.15	1.65	1.69
V′(C3,C4)						1.62	1.68
V(C4,C5)	1.66	3.24	3.23	3.11	2.53	2.37	2.21
V′(C4,C5)	1.7						
V(C1,C5)	2.01	2.19	2.21	2.39	3.21	1.63	1.7
V′(C1,C5)						1.7	1.74
V(C1,C6)	1.89	1.97	1.97	2.01	2.09	2.1	2.08

**Scheme 9:**
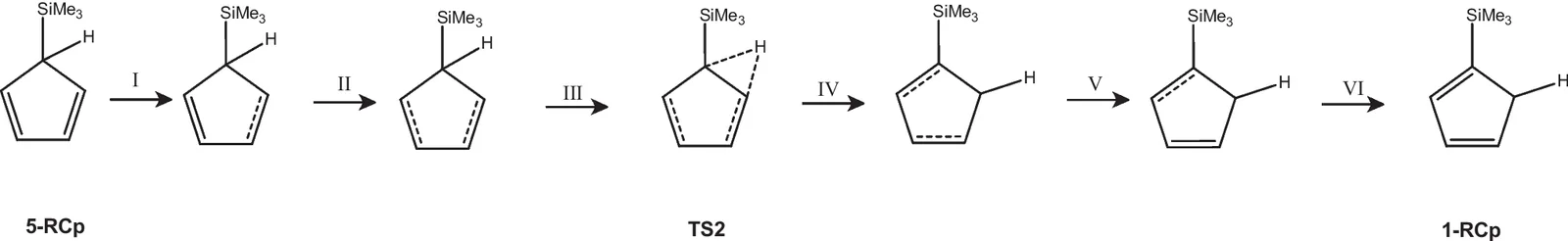
Simplified representation of the molecular mechanism of the isomerization reaction of SiMe_3_Cp using ELF-based Lewis structures

In contrast, the Si-substituted system (Scheme 9, Table 13) exhibits a slightly reduced population of the trisynaptic basin (≈1.63 e), accompanied by systematically lower maximum populations of the adjacent C–C disynaptic basins. This behavior suggests a partial attenuation of π-delocalization during the proton transfer, consistent with σ-π hyperconjugative interactions involving the C–Si bond that stabilize alternative electronic configurations along the reaction coordinate.

**Table 13:** BET analysis of the [1, 5]-sigmatropic hydrogen shift between C1 and C2 in SiMe_3_Cp along the intrinsic reaction coordinate (IRC) pathway. The BET phases were defined based on the changes in electron populations and molecular topology observed along the reaction coordinate. Distances are given in angstroms (Å) and electron populations in average number of electrons (e)

Phase	1	2	3	4	5	6	
d1(C1-H)	1.127	1.162	1.167	1.217	1.490	1.743	2.182
d2(C2-H)	2.128	1.667	1.643	1.478	1.210	1.148	1.120
V(C2,C3)	1.69	3.23	3.22	3.1	2.34	2.14	2.0
V′(C2,C3)	1.64						
V(C1,C2)	2.02	1.95	1.95	1.89	1.86	1.91	1.93
V(C1,H)	1.9	1.69	1.68				
V(C1,C2,H)				1.63			
V(C2,H)					1.65	1.76	1.97
V(C3,C4)	2.26	2.4	2.42	2.55	3.09	1.62	1.67
V′(C3,C4)						1.61	1.67
V(C4,C5)	1.69	1.63	3.21	3.12	2.57	2.38	2.23
V′(C4,C5)	1.64	1.6					
V(C1,C5)	2.01	2.16	2.17	2.29	2.91	3.11	1.63
V′(C1,C5)							1.59
V(C1,Si)	1.98	2.16	2.17	2.24	2.48	2.43	2.4

**Scheme 10:**
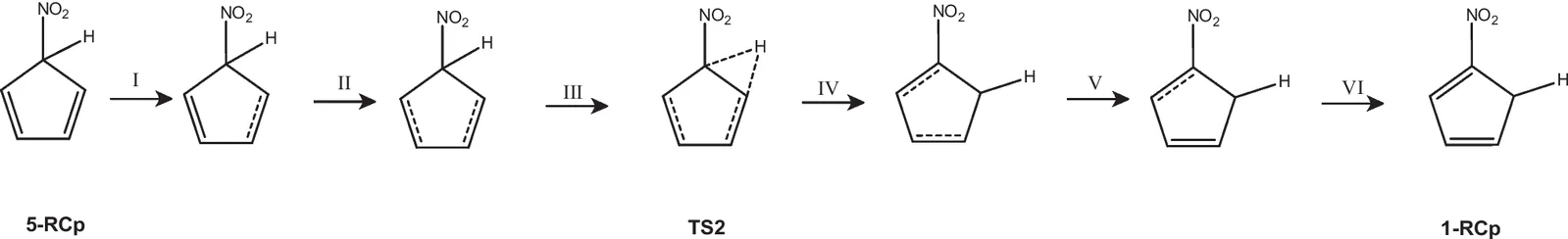
Simplified representation of the molecular mechanism of the isomerization reaction of NO_2_Cp using ELF-based Lewis structures

The NO_2_-substituted system (Scheme 10, Table 14) stands out as qualitatively distinct. Here, the trisynaptic basin reaches the highest population among all systems (≈1.76 e), indicating a more pronounced electron sharing between C1, C2, and H in the transition region. Simultaneously, the maximum population of the reorganized C–C basin increases up to 3.40 e, reflecting enhanced localization of electron density within specific C–C bonds. The persistent presence of monosynaptic basins V(N) and V′(N) further confirms that the strongly electron-withdrawing nitro group actively participates in the electronic reorganization by stabilizing charge separation and polarizing the π-framework during proton migration.

**Table 14:** BET analysis of the [1, 5]-sigmatropic hydrogen shift between C1 and C2 in NO_2_Cp along the intrinsic reaction coordinate (IRC) pathway. The BET phases were defined based on the changes in electron populations and molecular topology observed along the reaction coordinate. Distances are given in angstroms (Å) and electron populations in average number of electrons (e)

Phase	1	2	3	4	5	6	
d1(C1-H)	1.124	1.168	1.182	1.233	1.488	1.785	2.168
d2(C2-H)	2.132	1.605	1.558	1.446	1.220	1.146	1.121
V(C2,C3)	1.69	1.62	3.18	3.06	2.35	2.12	2.0
V′(C2,C3)	1.68	1.6					
V(C1,C2)	2.04	2.02	2.02	1.99	2.05	2.08	2.08
V(C1,H)	1.98	1.77	1.76				
V(C1,C2,H)				1.76			
V(C2,H)					1.58	1.75	1.95
V(C3,C4)	2.18	2.41	2.45	2.56	3.02	1.59	1.65
V′(C3,C4)						1.6	1.65
V(C4,C5)	1.69	3.19	3.17	3.08	2.62	2.39	2.25
V′(C4,C5)	1.68						
V(C1,C5)	2.05	2.27	2.31	2.42	3.26	3.4	1.71
V′(C1,C5)							1.77
V(C1,N)	2.2	2.24	2.24	2.27	2.34	2.38	2.38
V(N)	0.21	0.19	0.18	0.18	0.18	0.18	0.18
V′(N)				0.09	0.18	0.16	0.16

**Scheme 11:**
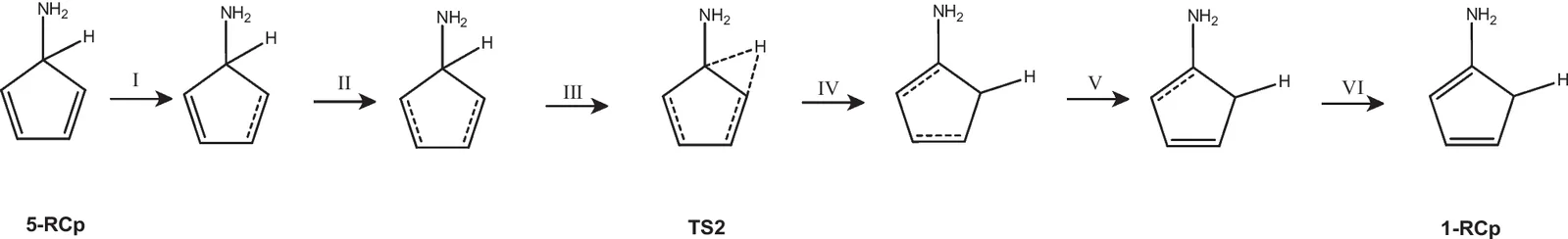
Simplified representation of the molecular mechanism of the isomerization reaction of NH_2_Cp using ELF-based Lewis structures

The NH_2_-substituted system (Table 15, Scheme 11) exhibits an intermediate behavior between the alkyl/silyl derivatives and the strongly electron-withdrawing NO_2_ system. The population of the trisynaptic basin V(C1,C2,H) reaches a value of approximately 1.68 e, similar to that observed for the Me and tBu derivatives, indicating a comparable degree of electron sharing in the transition region. At the same time, the maximum populations of the reorganized C–C disynaptic basins (e.g., V(C2,C3), V(C3,C5), or V(C4,C5)) remain close to ~ 3.2 e, reflecting efficient delocalization within the π-system. Unlike the NO_2_-substituted system, no pronounced enhancement of basin populations or extended persistence of auxiliary basins is observed, indicating a more synchronous redistribution of electron density. The relatively stable populations of the V(C1,N) and V(N) basins further suggest that the NH_2_ group does not actively participate in the bonding reorganization, but its electron-donating character slightly favors localization of electron density within the ring compared to the electron-withdrawing case.

**Table 15:** BET analysis of the [1, 5]-sigmatropic hydrogen shift between C1 and C2 in NH_2_Cp along the intrinsic reaction coordinate (IRC) pathway. The BET phases were defined based on the changes in electron populations and molecular topology observed along the reaction coordinate. Distances are given in angstroms (Å) and electron populations in average number of electrons (e)

Phases	1	2	3	4	5	6	7	
d1(C1-H)	1.12	1.131	1.165	1.226	1.473	1.726	1.748	2.164
d2(C2-H)	2.154	1.894	1.6	1.443	1.215	1.147	1.144	1.125
V(C1,C2)	1.97	1.96	1.94	1.9	1.92	1.95	1.95	1.98
V(C1,H)	1.95	1.84	1.72					
V(C1,C2,H)				1.68				
V(C2,H)					1.66	1.77	1.78	1.95
V(C3,C4)	2.06	2.15	2.3	2.46	3.23	1.74	1.76	1.81
V′(C3,C4)						1.69	1.67	1.74
V(C2,C3)	1.7	1.71	3.27	3.16	2.37	2.15	2.14	1.99
V′(C2,C3)	1.71	1.68						
V(C1,C5)	2.22	2.29	2.43	2.58	3.12	3.26	1.64	1.7
V′(C1,C5)							1.64	1.69
V(C4,C5)	1.8	3.44	3.31	3.18	2.65	2.44	2.42	2.27
V′(C4,C5)	1.7							
V(C1,N)	1.99	1.94	1.91	1.89	1.87	1.88	1.88	1.96
V(N)	1.79	1.87	1.91	1.93	1.95	1.93	1.93	1.83

Overall, the BET analysis demonstrates that while the proton transfer mechanism remains topologically conserved across the series, the substituent exerts a decisive influence on the degree of electron sharing in the transition region and on the redistribution of bonding electron density within the carbon skeleton. Electron-donating alkyl substituents favor a more delocalized and synchronous process, whereas the electron-withdrawing NO_2_ group induces a more polarized and electronically differentiated reaction pathway. The NH_2_ substituent follows the same general trend as electron-donating groups, while exhibiting an intermediate behavior that highlights the gradual transition between fully delocalized and strongly polarized regimes.

##### Proton migration from C2 to C3 in substituted cyclopentadienes

In the next stage of the study, the proton transfer reactions between the C2 and C3 carbon atoms of the cyclopentadienyl ring were analyzed. Particular attention was paid to the evolution of the bonding pattern along the reaction coordinate in order to determine how substituents influence the mechanism of the proton shift.

**Scheme 12:**
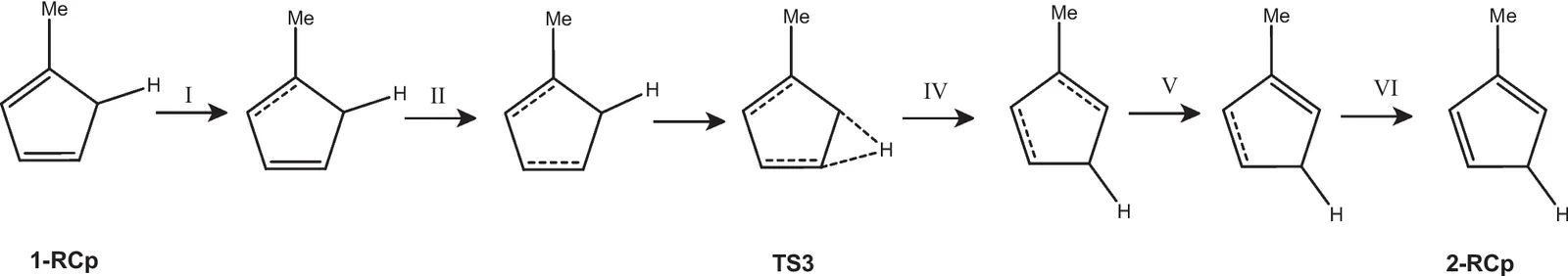
Simplified representation of the molecular mechanism of the isomerization reaction of MeCp using ELF-based Lewis structures

**Scheme 13:**
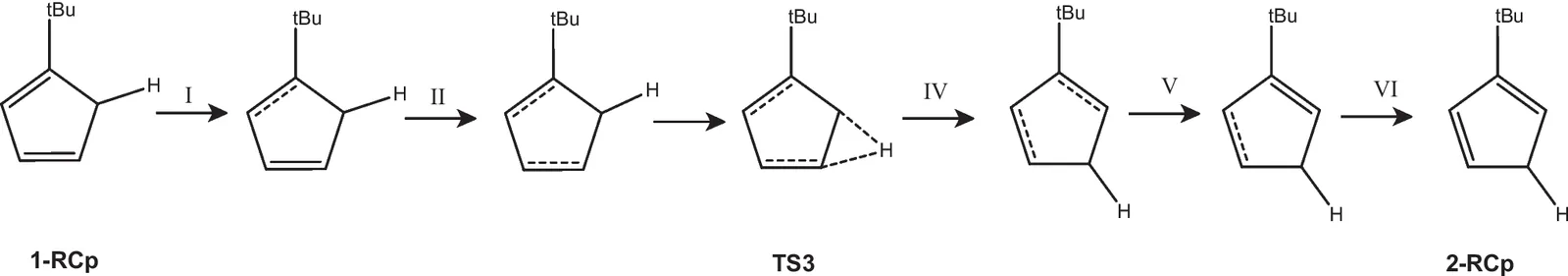
Simplified representation of the molecular mechanism of the isomerization reaction of tBuCp using ELF-based Lewis structures

For MeCp (Table 16, Scheme 12), the trisynaptic basin V(C2,C3,H) appears in phase 4 with a population of 1.66 e, confirming the three-center character of the proton transfer. The same bonding evolution is observed for tBuCp (Table 17, Scheme 13), where the population of the transient basin is 1.67 e, indicating that the alkyl substituent does not significantly influence the topology of the hydrogen shift.

**Table 16:** BET analysis of the [1, 5]-sigmatropic hydrogen shift between C2 and C3 in MeCp along the intrinsic reaction coordinate (IRC) pathway. The BET phases were defined based on the changes in electron populations and molecular topology observed along the reaction coordinate. Distances are given in angstroms (Å) and electron populations in average number of electrons (e)

Phase	1	2	3	4	5	6	7	
d1(C2-H)	1.122	1.152	1.169	1.226	1.485	1.668	1.714	2.146
d2(C3-H)	2.185	1.708	1.616	1.458	1.213	1.158	1.151	1.124
V(C2,C3)	1.99	1.98	1.97	1.93	1.94	1.98	1.98	1.99
V(C2,H)	1.99	1.75	1.7					
V(C2,C3,H)				1.66				
V(C3,H)					1.66	1.73	1.75	1.94
V(C1,C2)	2.02	2.19	2.25	2.41	3.18	1.67	1.71	1.72
V′(C1,C2)						1.64	1.62	1.72
V(C3,C4)	1.65	1.69	3.23	3.1	2.34	2.18	2.15	1.99
V′(C3,C4)	1.73	1.59						
V(C4,C5)	2.22	2.37	2.44	2.58	3.13	3.23	1.61	1.69
V′(C4,C5)							1.64	1.68
V(C1,C5)	1.77	3.3	3.26	3.15	2.59	2.44	2.41	2.24
V′(C1,C5)	1.68							
V(C1,C6)	2.03	2.02	2.02	2.01	2.0	2.0	2.0	2.01

**Table 17:** BET analysis of the [1, 5]-sigmatropic hydrogen shift between C2 and C3 in tBuCp along the intrinsic reaction coordinate (IRC) pathway. The BET phases were defined based on the changes in electron populations and molecular topology observed along the reaction coordinate. Distances are given in angstroms (Å) and electron populations in average number of electrons (e)

Phase	1	2	3	4	5	6	7	
d1(C2-H)	1.125	1.151	1.167	1.222	1.499	1.613	1.705	2.145
d2(C3-H)	2.146	1.717	1.624	1.466	1.206	1.170	1.152	1.123
V(C2,C3)	1.99	1.98	1.98	1.93	1.95	1.97	1.98	1.99
V(C2,H)	1.94	1.75	1.71					
V(C2,C3,H)				1.67				
V(C3,H)					1.66	1.7	1.74	1.94
V(C1,C2)	2.04	2.21	2.26	2.44	3.19	1.67	1.69	1.71
V′(C1,C2)						1.61	1.64	1.72
V(C3,C4)	1.69	1.59	3.22	3.09	2.32	2.22	2.15	1.99
V′(C3,C4)	1.68	1.69						
V(C4,C5)	2.22	2.38	2.44	2.59	3.15	3.22	1.62	1.7
V′(C4,C5)							1.65	1.68
V(C1,C5)	1.69	3.28	3.24	3.11	2.58	2.47	2.41	2.24
V′(C1,C5)	1.73							
V(C1,C6)	2.08	2.08	2.08	2.07	2.05	2.05	2.05	2.07

**Scheme 14:**
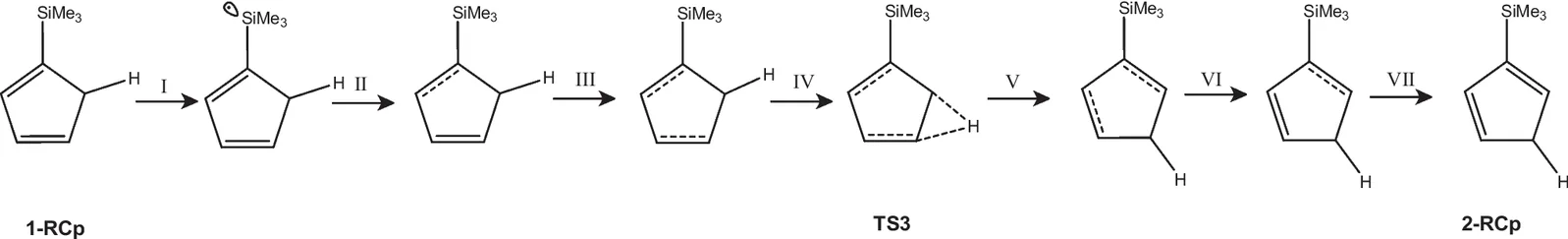
Simplified representation of the molecular mechanism of the isomerization reaction of SiMe_3_Cp using ELF-based Lewis structures

The SiMe_3_Cp derivative (Table 18, Scheme 14) follows the same mechanism; however, a slightly lower population of the trisynaptic basin (1.66 e) and changes in neighboring C–C basins indicate minor differences in electron redistribution associated with the σ–π interactions involving the Si–C bond. Nevertheless, the V(C1,Si) basin remains essentially unchanged, confirming that the Si–C bond is not directly involved in the migration process.

**Table 18:** BET analysis of the [1, 5]-sigmatropic hydrogen shift between C2 and C3 in SiMe_3_Cp along the intrinsic reaction coordinate (IRC) pathway. The BET phases were defined based on the changes in electron populations and molecular topology observed along the reaction coordinate. Distances are given in angstroms (Å) and electron populations in average number of electrons (e)

Phase	1	2	3	4	5	6	7	8	9	
d1(C2-H)	1.123	1.131	1.131	1.138	1.153	1.215	1.503	1.686	1.887	2.141
d2(C3-H)	2.146	2.019	2.003	1.868	1.713	1.484	1.206	1.157	1.136	1.125
V(C2,C3)	2.0	2.01	2.02	2.01	2.0	1.96	1.96	2.0	2.0	2.0
V(C2,H)	1.94	1.88	1.87	1.81	1.74					
V(C2,C3,H)						1.66				
V(C3,H)							1.65	1.73	1.82	1.94
V(C1,C2)	1.93	1.96	1.96	2.01	2.09	2.26	2.97	3.11	1.59	1.63
V′(C1,C2)									1.61	1.62
V(C3,C4)	1.67	1.64	1.64	1.6	3.22	3.06	2.3	2.16	2.06	1.99
V′(C3,C4)	1.68	1.68	1.68	1.69						
V(C4,C5)	2.23	2.26	2.27	2.32	2.4	2.59	3.16	1.59	1.67	1.68
V′(C4,C5)								1.67	1.66	1.7
V(C1,C5)	1.59	1.6	1.6	3.11	3.05	2.89	2.42	2.3	2.21	2.15
V′(C1,C5)	1.63	1.58	1.58							
V(C1,Si)	2.4	2.4	2.42	2.44	2.45	2.49	2.43	2.37	2.34	2.32
V(Si)		0.34

**Scheme 15:**
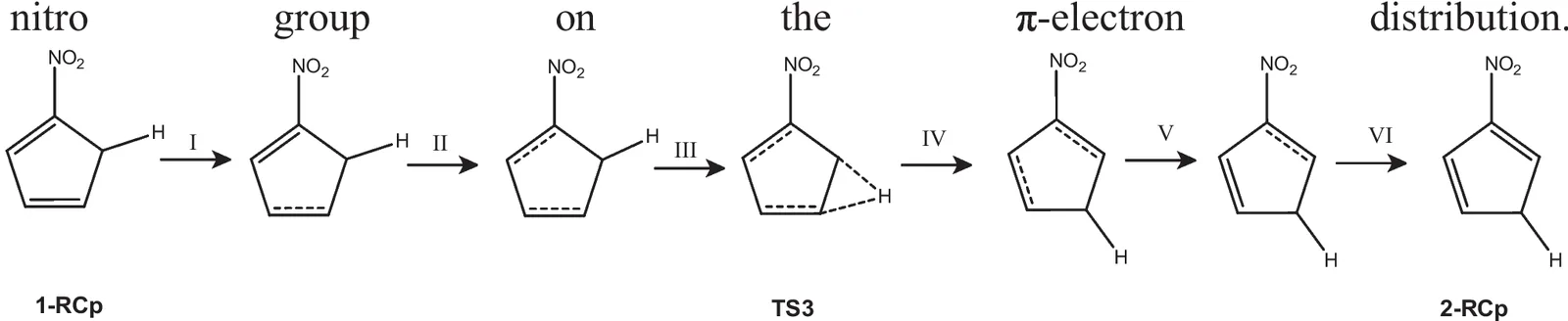
Simplified representation of the molecular mechanism of the isomerization reaction of NO_2_Cp using ELF-based Lewis structures

The NO_2_Cp system (Table 19, Scheme 15) shows the most pronounced electronic differentiation. The transient V(C2,C3,H) basin has a slightly lower population (1.64 e), but the substituent induces larger variations in the neighboring C–C basins and maintains the V(C1,N) basin throughout the reaction coordinate, reflecting the influence of the electron-withdrawing nitro group on the π-electron distribution.

**Table 19:** BET analysis of the [1, 5]-sigmatropic hydrogen shift between C2 and C3 in NO_2_Cp along the intrinsic reaction coordinate (IRC) pathway. The BET phases were defined based on the changes in electron populations and molecular topology observed along the reaction coordinate. Distances are given in angstroms (Å) and electron populations in average number of electrons (e)

Phase	1	2	3	4	5	6	7	
d1(C2-H)	1.123	1.146	1.153	1.212	1.510	1.576	1.780	2.136
d2(C3-H)	2.141	1.771	1.727	1.502	1.211	1.188	1.147	1.122
V(C2,C3)	1.99	2.01	2.01	1.98	1.99	2.02	2.03	2.01
V(C2,H)	1.93	1.76	1.73					
V(C2,C3,H)				1.64				
V(C3,H)					1.64	1.64	1.75	1.94
V(C1,C2)	2.08	2.18	2.2	2.36	3.26	3.31	1.74	1.73
V′(C1,C2)							1.66	1.72
V(C3,C4)	1.65	3.18	3.16	2.99	2.27	2.22	2.1	2.0
V′(C3,C4)	1.64							
V(C4,C5)	2.25	2.4	2.43	2.62	3.16	1.56	1.65	1.65
V′(C4,C5)						1.64	1.63	1.7
V(C1,C5)	1.74	1.69	3.38	3.28	2.56	2.5	2.39	2.28
V′(C1,C5)	1.74	1.71						
V(C1,N)	2.37	2.37	2.37	2.36	2.35	2.35	2.34	2.33
V(N)	0.18	0.18	0.18	0.17	0.17	0.17	0.17	0.17
V′(N)	0.17	0.16	0.16	0.16	0.16	0.15	0.16	0.19

**Scheme 16:**
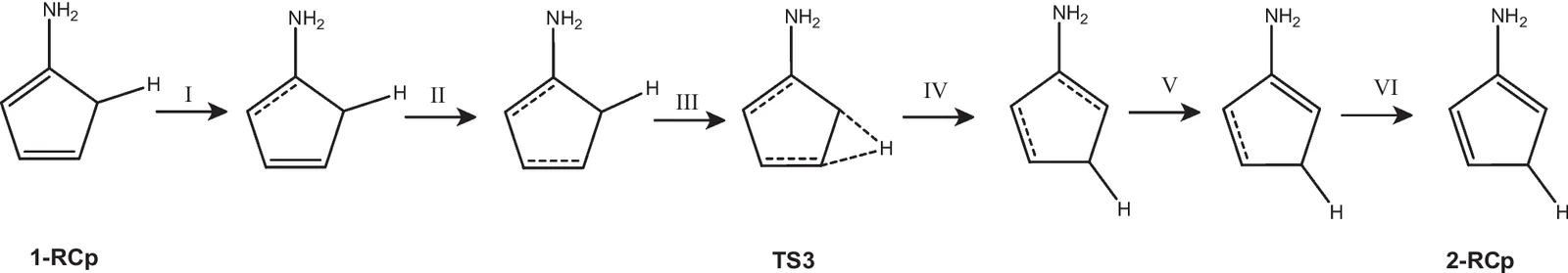
Simplified representation of the molecular mechanism of the isomerization reaction of NH_2_Cp using ELF-based Lewis structures

In contrast, NH_2_Cp (Table 20, Scheme 16) exhibits a bonding evolution comparable to alkyl derivatives. The population of V(C2,C3,H) reaches 1.68 e, while the nearly constant populations of V(C1,N) and V(N) indicate that the amino group modulates the electron density distribution but is not directly involved in proton migration.

**Table 20:** BET analysis of the [1, 5]-sigmatropic hydrogen shift between C2 and C3 in NH_2_Cp along the intrinsic reaction coordinate (IRC) pathway. The BET phases were defined based on the changes in electron populations and molecular topology observed along the reaction coordinate. Distances are given in angstroms (Å) and electron populations in average number of electrons (e)

Phases	1	2	3	4	5	6	7	
d1(C2-H)	1.12	1.131	1.165	1.226	1.473	1.726	1.748	2.164
d2(C3-H)	2.154	1.894	1.6	1.443	1.215	1.147	1.144	1.125
V(C2,C3)	1.97	1.96	1.94	1.9	1.92	1.95	1.95	1.98
V(C2,H)	1.95	1.84	1.72					
V(C2,C3,H)				1.68				
V(C3,H)					1.66	1.77	1.78	1.95
V(C1,C2)	2.06	2.15	2.3	2.46	3.23	1.74	1.76	1.81
V′(C1,C2)						1.69	1.67	1.74
V(C3,C4)	1.7	1.71	3.27	3.16	2.37	2.15	2.14	1.99
V′(C3,C4)	1.71	1.68						
V(C4,C5)	2.22	2.29	2.43	2.58	3.12	3.26	1.64	1.7
V′(C4,C5)							1.64	1.69
V(C1,C5)	1.8	3.44	3.31	3.18	2.65	2.44	2.42	2.27
V′(C1,C5)	1.7							
V(C1,N)	1.99	1.94	1.91	1.89	1.87	1.88	1.88	1.96
V(N)	1.79	1.87	1.91	1.93	1.95	1.93	1.93	1.83

Overall, the comparative BET analysis demonstrates that proton migration from C2 to C3 follows the same non-classical mechanism for all substituted cyclopentadienes. The substituents mainly affect the extent of electron redistribution rather than the reaction topology. Electron-donating groups (Me, tBu, NH_2_) favor a more delocalized bonding pattern, whereas NO2 induces a more polarized electronic structure. The SiMe_3_ group exhibits only minor deviations related to σ–π interactions, without changing the fundamental migration pathway.

##### Proton migration from C3 to C4 in substituted cyclopentadienes

**Scheme 17:**
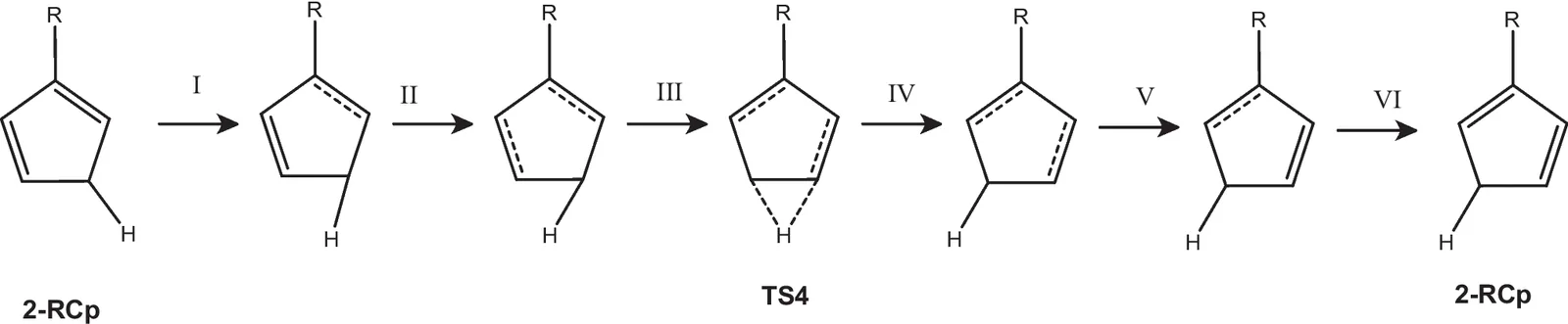
Simplified representation of the molecular mechanism of the isomerization reaction of RCp using ELF-based Lewis structures

In the next stage of the study, we investigated the proton isomerization from the C3 to the C4 carbon atom in substituted cyclopentadiene derivatives. All substituted cyclopentadiene derivatives undergo hydrogen migration via the same non-classical BET mechanism (Scheme 17) involving a transient trisynaptic V(C3,C4,H) basin. Alkyl and silyl substituents promote a compact and synchronous bonding evolution, characterized by short-lived auxiliary basins and rapid stabilization of the C4-H bond (Tables 21, 22, 23, 24, and 25). In contrast, the strongly electron-withdrawing NO_2_ group induces a pronounced asynchronicity of the process (Table 24), manifested by delayed appearance of the trisynaptic basin, prolonged persistence of auxiliary V′ basins, and enhanced redistribution of electron density within the carbon framework. These results demonstrate that substituent effects in this isomerization are predominantly quantitative rather than mechanistic, with NO_2_ uniquely amplifying the degree of electronic reorganization along the reaction coordinate.

**Table 21:** BET analysis of the [1, 5]-sigmatropic hydrogen shift between C3 and C4 in MeCp along the intrinsic reaction coordinate (IRC) pathway. The BET phases were defined based on the changes in electron populations and molecular topology observed along the reaction coordinate. Distances are given in angstroms (Å) and electron populations in average number of electrons (e)

Phase	1	2	3	4	5	6	7	
d1(C3-H)	1.122	1.153	1.161	1.218	1.507	1.688	1.734	2.146
d2(C4-H)	2.146	1.711	1.666	1.485	1.209	1.157	1.150	1.122
V(C3,C4)	1.99	1.99	1.99	1.94	1.96	1.98	1.99	1.99
V(C3,H)	1.95	1.75	1.72					
V(C3,C4,H)				1.66				
V(C4,H1)					1.66	1.74	1.76	1.95
V(C4,C5)	1.7	1.68	3.24	3.12	2.3	2.15	2.12	1.99
V′(C4,C5)	1.67	1.6						
V(C2,C3)	1.99	2.13	2.16	2.32	3.14	1.63	1.7	1.7
V′(C2,C3)						1.63	1.59	1.67
V(C1,C2)	1.74	3.31	3.29	3.19	2.56	2.42	2.39	2.24
V′(C1,C2)	1.69							
V(C1,C5)	2.24	2.4	2.43	2.59	3.2	3.3	1.69	1.74
V′(C1,C5)							1.64	1.69

**Table 22:** BET analysis of the [1, 5]-sigmatropic hydrogen shift between C3 and C4 in tBuCp along the intrinsic reaction coordinate (IRC) pathway. The BET phases were defined based on the changes in electron populations and molecular topology observed along the reaction coordinate. Distances are given in angstroms (Å) and electron populations in average number of electrons (e)

Phase	1	2	3	4	5	6	7	
d1(C3-H)	1.123	1.152	1.159	1.223	1.496	1.654	1.700	2.147
d2(C4-H)	2.140	1.721	1.676	1.472	1.212	1.163	1.155	1.125
V(C3,C4)	1.99	1.99	1.99	1.95	1.96	1.99	1.99	1.99
V(C3,H)	1.94	1.75	1.73					
V(C3,C4,H)				1.66				
V(C4,H)					1.66	1.72	1.74	1.95
V(C2,C3)	2.0	2.15	2.18	2.36	3.15	1.61	1.69	1.68
V′(C2,C3)						1.65	1.58	1.7
V(C4,C5)	1.66	1.67	3.24	3.08	2.3	2.17	2.14	1.99
V′(C4,C5)	1.7	1.6						
V(C1,C5)	2.26	2.43	2.45	2.65	3.21	3.29	1.66	1.71
V′(C1,C5)							1.66	1.72
V(C1,C2)	1.71	3.28	3.26	3.14	2.57	2.44	2.41	2.24
V′(C1,C2)	1.71							
V(C1,C6)	2.06	2.06	2.06	2.06	2.06	2.06	2.06	2.06

**Table 23:** BET analysis of the [1, 5]-sigmatropic hydrogen shift between C3 and C4 in SiMe_3_Cp along the intrinsic reaction coordinate (IRC) pathway. The BET phases were defined based on the changes in electron populations and molecular topology observed along the reaction coordinate. Distances are given in angstroms (Å) and electron populations in average number of electrons (e)

Phase	1	2	3	4	5	6	7	
d1(C3-H)	1.123	1.139	1.159	1.213	1.502	1.662	1.865	2.148
d2(C4-H)	2.145	1.847	1.666	1.484	1.206	1.160	1.137	1.123
V(C3,C4)	2.0	1.99	1.99	1.94	1.95	1.98	1.99	2.0
V(C3,H)	1.94	1.8	1.72					
V(C3,C4,H)				1.65				
V(C4,H)					1.65	1.71	1.81	1.94
V(C2,C3)	1.99	2.1	2.19	2.34	3.14	1.6	1.7	1.69
V′(C2,C3)						1.65	1.62	1.68
V(C4,C5)	1.67	1.7	3.24	3.12	2.32	2.19	2.08	2.0
V′(C4,C5)	1.7	1.62						
V(C1,C5)	2.15	2.25	2.34	2.48	3.03	3.12	1.6	1.62
V′(C1,C5)							1.58	1.63
V(C1,C2)	1.63	3.17	3.11	3.01	2.45	2.33	2.24	2.15
V′(C1,C2)	1.62							
V(C1,Si)	2.33	2.33	2.33	2.34	2.33	2.33	2.32	2.32

**Table 24:** BET analysis of the [1, 5]-sigmatropic hydrogen shift between C3 and C4 in NO_2_Cp along the intrinsic reaction coordinate (IRC) pathway. The BET phases were defined based on the changes in electron populations and molecular topology observed along the reaction coordinate. Distances are given in angstroms (Å) and electron populations in average number of electrons (e)

Phase	1	2	3	4	5	6	7	8	
d1(C3-H)	1.121	1.156	1.181	1.213	1.503	1.595	1.665	1.711	2.186
d2(C4-H)	2.186	1.687	1.572	1.481	1.204	1.175	1.160	1.153	1.121
V(C3,C4)	1.99	1.98	1.98	1.94	1.95	1.98	1.98	1.98	1.99
V(C3,H)	1.96	1.71	1.65						
V(C3,C4,H)				1.64					
V(C4,H)					1.63	1.66	1.7	1.72	1.96
V(C2,C3)	2.02	2.19	2.27	2.37	3.12	1.63	1.66	1.67	1.7
V′(C2,C3)						1.57	1.57	1.58	1.65
V(C4,C5)	1.7	1.67	3.17	3.09	2.34	2.25	2.2	2.18	2.02
V′(C4,C5)	1.66	1.57							
V(C1,C5)	2.29	2.46	2.54	2.63	3.24	3.29	3.32	1.62	1.7
V′(C1,C5)								1.73	1.77
V(C1,C2)	1.7	3.33	3.29	3.22	2.6	2.52	2.48	2.45	2.29
V′(C1,C2)	1.77								
V(C1,N)	2.33	2.34	2.33	2.33	2.33	2.33	2.34	2.33	2.33
V(N)	0.18	0.19	0.18	0.18	0.19	0.18	0.18	0.18	0.18
V′(N)	0.17	0.17	0.16	0.17	0.16	0.15	0.17	0.17	0.17

**Table 25:** BET analysis of the [1, 5]-sigmatropic hydrogen shift between C3 and C4 in NH_2_Cp along the intrinsic reaction coordinate (IRC) pathway. The BET phases were defined based on the changes in electron populations and molecular topology observed along the reaction coordinate. Distances are given in angstroms (Å) and electron populations in average number of electrons (e)

Phases	1	2	3	4	5	6	7	
d1(C3-H)	1.125	1.139	1.171	1.227	1.512	1.667	1.887	2.143
d2(C4-H)	2.144	1.866	1.645	1.492	1.217	1.166	1.137	1.125
V(C2,C4)	1.99	2	2	1.97	1.98	2	2	1.99
V(C3,H4)	1.95	1.83	1.72					
V(C3,C4,H)				1.67				
V(C4,H)					1.66	1.73	1.84	1.95
V(C1,C2)	1.7	1.69	3.26	3.18	2.29	2.16	2.05	1.98
V′(C1,C2)	1.68	1.65						
V(C2,C3)	1.98	2.06	2.18	2.32	3.2	1.63	1.69	1.7
V′(C2,C3)						1.66	1.66	1.68
V(C4,C5)	1.74	3.47	3.38	3.27	2.56	2.44	2.33	2.27
V′(C4,C5)	1.81							
V(C1,C5)	2.27	2.35	2.46	2.58	3.29	3.39	1.75	1.81
V′(C1,C5)							1.73	1.74
V(C1,N)	1.96	1.95	1.94	1.94	1.94	1.95	1.95	1.96
V(N)	1.83	1.86	1.87	1.88	1.88	1.87	1.86	1.83

For the alkyl-substituted derivatives MeCp and tBuCp (Tables 21 and 22), the reaction follows a highly similar bonding evolution. The population of the initial V(C3,H) basin decreases from approximately 1.95 e to 1.72–1.73 e, while the newly formed V(C4,H) basin reaches about 1.95 e at the final stages of the reaction. The population of the V(C3,C4) basin remains nearly constant (~ 1.94–2.00 e), indicating preservation of the carbon framework during hydrogen migration. The formation of the trisynaptic basin V(C3,C4,H) with a population of approximately 1.66 e reflects a compact and synchronous electron redistribution along the reaction coordinate.

A similar mechanism is observed for SiMe_3_Cp (Table 23). The transient V(C3,C4,H) basin reaches a population of approximately 1.65 e, followed by the formation of the V(C4,H) basin with a final population of 1.94 e. The nearly unchanged population of the V(C1,Si) basin (~ 2.32–2.34 e) indicates that the Si–C bond remains unaffected during the proton transfer, confirming that the silyl substituent influences the process mainly through electronic effects rather than direct participation in the bonding rearrangement.

In contrast, the NO_2_-substituted derivative exhibits a more complex redistribution of electron density (Table 24). Although the same fundamental BET sequence is observed, the appearance of the trisynaptic basin occurs at a slightly later stage of the reaction coordinate and is accompanied by more pronounced fluctuations in the neighboring C–C basins. Additionally, auxiliary basins V′ persist for a longer portion of the reaction coordinate, indicating a more extensive reorganization of electron density within the cyclopentadienyl ring. The presence of persistent monosynaptic basins associated with the nitrogen atom reflects the strong electron-withdrawing character of the NO_2_ group, which enhances polarization of the π-electron system and leads to a less synchronous bonding evolution.

The NH_2_-substituted derivative (Table 25) follows the same non-classical pathway as the other systems. The transient V(C3,C4,H) basin reaches approximately 1.67 e, while the final V(C4,H) basin increases to about 1.95 e. The populations of V(C1,N) and V(N) remain essentially constant, indicating that the amino group is not directly involved in the proton transfer but modulates electron density distribution through its electron-donating character.

Overall, the comparative BET analysis demonstrates that the C3–C4 proton migration proceeds through an identical topological mechanism in all substituted cyclopentadienes. The substituents affect mainly the extent and synchronization of electron redistribution rather than the reaction pathway itself. Alkyl and silyl groups promote a compact and relatively synchronous bonding evolution, whereas the strongly electron-withdrawing NO_2_ group increases polarization and enhances electronic reorganization within the ring system. The NH_2_ substituent exhibits intermediate behavior, maintaining the same mechanism while favoring a more localized redistribution of electron density.

### QTAIM analysis of electron density and bonding interactions

To complement the topological analysis of the BET and to clarify the bonding pattern between the cyclopentadienyl ring and the R substituent, the topology of the electron density distribution was examined within the framework of QTAIM [18, 19]. The assessment of the sign of the Laplacian of the electron density, together with other descriptors evaluated at the bond critical points (BCPs), such as the electron density (*ρ*), the Lagrangian kinetic energy (*G*), and the local energy density (*H*), provides valuable insight into the nature of the interactions they characterize [20]. Key bonding parameters were evaluated for the transition state (TS1), selected points along the IRC, and the final product (RCp) for each substituted cyclopentadienyl system (Table 26).

**Table 26:** QTAIM topological indicators at the BCP associated with the R-Cp region. *ρ* is given in e·Å^−3^, *∇*^2^*ρ* in e·Å^−5^, and *H*, *G,* and *V* in hartree·Å^−3^

		***ρ*(*r*)**	***∇***^**2**^***ρ*(*r*)**	***H*(*r*)**	***V*(*r*)**	***G*(*r*)**	**Sign(*λ***_**2**_**)*ρ***	**Ellipticity**	**|*V*|/*G***	***G*/*ρ***
tBu	TS1	0.742	0.486	−0.169	−0.459	0.290	−0.742	3.23	1.58	0.39
	IRC 10	0.905	−0.144	−0.303	−0.571	0.267	−0.905	0.418	2.14	0.30
	IRC 52	0.216	−0.428	−0.157	−0.208	0.504	−0.216	0.006	0.41	2.33
	RCp	0.221	−0.449	−0.164	−0.215	0.514	−0.221	0.008	0.42	2.33
Me	TS1	0.106	0.050	−0.036	−0.084	0.048	−0.106	46.9	1.75	0.45
	IRC 10	0.132	−0,936	−0,632	−0.103	0.397	−0.132	0.35	0.26	3.01
	IRC 48	0.224	−0.467	−0.171	−0.225	0.540	−0.224	0.005	0.42	2.41
	RCp	0.229	−0.488	−0.177	−0.232	0.549	−0.229	0.006	0.42	2.40
SiMe_3_	TS1	0.072	−0.019	−0.040	−0.761	0.356	−0.722	3.11	2.14	4.94
	IRC 10	0.08	0.02	−0.048	−0.102	0.533	−0.799	0.42	0.19	6.66
	IRC 54	0.105	0.158	−0.064	−0.168	0.104	−0.105	0.013	1.62	0.99
	RCp	0.106	0.166	−0.065	−0.171	0.106	−0.106	0.007	1.61	1.00
NO_2_	TS1	0.771	0.160	−0.114	−0.629	0.515	−0.771	14.40	1.22	0.67
	IRC 10	0.112	0.253	−0.430	−0.925	0.494	−0.112	0.31	1.87	4.41
	IRC 40	0.230	−0.512	−0.235	−0.342	0.107	−0.230	0.095	3.20	0.47
	RCp	0.235	−0.546	−0.247	−0.359	0.111	−0.235	0.083	3.23	0.47
NH_2_	TS1	0.186	−0.138	−0.154	−0.273	0.119	−0.186	0.967	2.29	0.64
	TS1	0.186	−0.134	−0.153	−0.272	0.119	−0.186	0.980	2.28	0.64
	IRC 10	0.213	−0.347	−0.203	−0.320	0.116	−0.213	0.398	2.75	0.55
	IRC 65	0.257	−0.648	−0.264	−0.367	0.102	−0.257	0.032	3.58	0.40
	RCp	0.260	−0.671	−0.272	−0.377	0.105	−0.260	0.026	3.60	0.40

The analysis of the transition states (TS1) reveals a striking differentiation in the bonding regimes across the studied substituents. For the SiMe_3_ group, the migration is facilitated by a robust covalent interaction that is maintained even at the energy maximum. This is evidenced by a negative Laplacian (*∇*^2^*ρ* = −0.019 a.u.) and a ∣*V*∣/*G* ratio of 2.14, which, according to the Cremer and Kraka criteria, signifies a shared-shell (purely covalent) interaction. This unique covalent stabilization at the TS likely accounts for the enhanced migratory aptitude of silyl groups compared to alkyl derivatives.

In contrast, the transition states for the methyl and nitro groups exhibit a fundamentally different topological signature. The methyl shift is characterized by a “delocalized” TS, where the extremely high ellipticity (*ϵ* = 46.9) and a positive Laplacian (*∇*^2^*ρ* = 0.050 a.u.) indicate a highly unstable and diffuse bond path. For the NO_2_ group, the interaction at the TS is predominantly polar/electrostatic in nature, as suggested by the low ∣*V*∣/*G* ratio (1.22) and a significant charge depletion at the BCP.

Furthermore, the evolution of the Laplacian along the IRC path highlights a “topological catastrophe” for the alkyl substituents. While the tBu and Me groups show positive *∇*^2^*ρ* values at the TS (transit/closed-shell regime), a rapid inversion to negative values occurs early in the IRC (e.g., *∇*^2^*ρ* = −0.936 for Me at IRC 10), marking the abrupt collapse of the delocalized transition state into a localized covalent bond. Interestingly, the tBu derivative shows a significant electronic compression at the TS (*ρ* = 0.742 a.u.), which is nearly three times higher than in the final 5-RCp product, reflecting the severe steric constraints imposed during the migration of the bulky tert-butyl moiety.

The QTAIM analysis reveals that although all systems converge to a classical covalent bond in the product, the nature of the transition state varies significantly with the substituent, ranging from highly delocalized (Me) to strongly polarized (NO_2_, SiMe_3_), while the NH_2_ group exhibits the most balanced and localized bonding character.

Representative QTAIM molecular graphs for the analyzed structures are provided in the Supporting Information (Figure S6-S10).

## Conclusions

The combined analysis of BET, QTAIM, and thermodynamic data provides a coherent picture of the R-shift in substituted cyclopentadienyl systems. For MeCp, BET analysis shows that the migrating methyl group remains covalently bound to the Cp ring throughout the process, which is corroborated by QTAIM parameters indicating a classical covalent bond. The thermodynamic barriers for MeCp are moderate (ΔG^‡^ ≈ 42–43 kcal/mol), consistent with a concerted, non-polarized migration.

In contrast, tBuCp, SiMe_3_Cp, and NO_2_Cp exhibit polarized bonding in the transition states, as indicated by QTAIM analysis (high |*V*|/*G* ratios and significant ellipticities), reflecting partial charge separation during migration. While the NH_2_ group exhibits the most balanced and localized bonding character. BET results confirm the formation of elongated, polarized bonds rather than full dissociation of the migrating group. Thermodynamically, these systems show slightly lower or comparable activation barriers (ΔG^‡^≈ 15–52 kcal/mol depending on substituent and solvent), with the lowest barrier observed for the SiMe_3_Cp derivative. In all cases, the formation of the final R-shifted products is exergonic. The correlation between bond polarization and lower transition-state stability suggests that electron-withdrawing or bulky substituents modulate the character and energetics of the migration.

Overall, the R-shift involves concerted migration with variable bond polarization, depending on the nature of the substituent: covalent for small alkyl groups (Me) and polarized for bulky (tBu, SiMe_3_), electron-withdrawing groups (NO_2_), or strongly electron-donating groups (NH_2_), the latter favoring a more localized electron density redistribution without pronounced trisynaptic delocalization.

The analysis of the proton migration processes in substituted cyclopentadiene derivatives reveals that both investigated hydrogen shifts proceed through the same fundamental non-classical BET mechanism. In all studied systems, the proton transfer occurs via the transient formation of a trisynaptic V(C,C,H) basin located in the transition-state region, indicating a concerted redistribution of electron density rather than complete proton dissociation. The subsequent disappearance of this basin and the formation of a new disynaptic C–H basin confirm the establishment of the final C–H bond. The analyzed reactions can formally be classified as [1, 5]-sigmatropic rearrangements, involving the migration of a substituent or hydrogen atom across the cyclopentadienyl framework.

The nature of the substituent primarily influences the degree of electronic reorganization along the reaction coordinate rather than the mechanism itself. Alkyl and silyl substituents (Me, tBu, and SiMe_3_) promote a compact and relatively synchronous bonding evolution, whereas the strongly electron-withdrawing NO_2_ group induces a more pronounced redistribution of electron density and a slightly increased asynchronicity of the proton migration. In contrast, the electron-donating NH_2_ group favors a more localized and less delocalized electron density redistribution, without significantly altering the overall reaction pattern. Despite these differences, the overall bonding pattern and topological sequence of the reaction remain consistent across all analyzed derivatives, demonstrating that substituent effects in these proton shifts are predominantly quantitative rather than mechanistic.

## Computational details

Quantum-chemical computations were carried out with the Gaussian 16 software suite [21]. Geometry optimizations and energy evaluations were carried out employing the ωB97X-D range-separated hybrid density functional [22], which includes empirical dispersion corrections, in combination with the 6–311+G(d,p) basis set [23–25]. The inclusion of diffuse and polarization functions was essential for an accurate description of the electronic structure of the cyclopentadiene derivatives and the corresponding transition states. Molecular geometries were visualized using GaussView 6.0 [26].

Thermodynamic and kinetic parameters were computed at the ωB97X-D/6–311+G(d,p) level of theory both in the gas phase and in solution. Solvent effects were accounted for using the polarizable continuum model (PCM) [27, 28]. Calculations were performed for solvents of different polarities, including toluene, acetone, and nitromethane, in order to evaluate the influence of the reaction medium on the energetic and mechanistic features of the isomerization processes.

All reactants, intermediates, and products were fully optimized using the Berny optimization algorithm [29, 30], ensuring convergence to stationary points on the potential energy surface. Transition-state geometries were initially located using the QST2 (quadratic synchronous transit) method and subsequently refined. Harmonic vibrational frequency calculations were performed [31] for all optimized structures to verify their character: minima were confirmed by the absence of imaginary frequencies, while each transition state displayed a single imaginary frequency corresponding to the reaction coordinate.

The identity of each transition state was further verified by visual inspection of the associated vibrational mode and by performing intrinsic reaction coordinate (IRC) calculations in both the forward and reverse directions, thereby confirming the correct connectivity between each transition state and its neighboring minima on the potential energy surface.

Topological analyses of the electron localization function (ELF) [31–33] were carried out using TopMod 09 [34] based on monodeterminantal wavefunctions, with a grid spacing of 0.1 atomic units. For the BET [17] studies, ELF topological analyses were performed along the intrinsic reaction coordinate (IRC). Additionally, QTAIM [18, 35] analyses were generated using Multiwfn software [19].

## Supplementary Information

Below is the link to the electronic supplementary material.

ESM 1 (DOCX 1.78 MB)

## Data Availability

The data that support the findings of this study are available from the corresponding author upon reasonable request.
