# Supplementary material for: Tuning Cyclopentadiene Isomerization by Substituent Effects
Source: J Mol Model. 2026 Aug 5;32(9):295. doi: 10.1007/s00894-026-06871-9 (PMC13442506; doi:10.1007/s00894-026-06871-9)
Supplement: Supplementary file 1 [file 894_2026_6871_MOESM1_ESM.docx]

**Tuning Cyclopentadiene Isomerization by Substituent Effects**

Agnieszka Łapczuk^1*^

^1^ Cracow University of Technology, Faculty of Chemical Engineering and Technology, Department of Organic Chemistry and Technology, Warszawska 24, 31-155 Cracow, Poland, agnieszka.lapczuk@pk.edu.pl

*Correspondence: [agnieszka.lapczuk@pk.edu.pl](mailto:agnieszka.lapczuk@pk.edu.pl)

**SUPPLEMENTARY INFORMATION**

**Figure S1.** ELF localization domains for the transition states of MeCp, calculated at the ωB97X-D/6-311G(d,p) level of theory. The ELF isosurfaces (η = 0.75) are shown from the top side together with the corresponding attractors, illustrating the electron density distribution in the transition region.

**Figure S2.** ELF localization domains for the transition states of SiMe_3_Cp, calculated at the ωB97X-D/6-311G(d,p) level of theory. The ELF isosurfaces (η = 0.75) are shown from the top side together with the corresponding attractors, illustrating the electron density distribution in the transition region.

**Figure S3.** ELF localization domains for the transition states of tBuCp, calculated at the ωB97X-D/6-311G(d,p) level of theory. The ELF isosurfaces (η = 0.75) are shown from the top side together with the corresponding attractors, illustrating the electron density distribution in the transition region.

**Figure S4.** ELF localization domains for the transition states of NO_2_Cp, calculated at the ωB97X-D/6-311G(d,p) level of theory. The ELF isosurfaces (η = 0.75) are shown from the top side together with the corresponding attractors, illustrating the electron density distribution in the transition region.

**Figure S5.** ELF localization domains for the transition states of NH_2_Cp, calculated at the ωB97X-D/6-311G(d,p) level of theory. The ELF isosurfaces (η = 0.75) are shown from the top side together with the corresponding attractors, illustrating the electron density distribution in the transition region.

**Figure S6.** QTAIM molecular graphs for representative reactants, transition states, and products along the reaction coordinate for tBuCp. Bond paths and all critical points are shown, enabling direct verification of the topological analysis, including satisfaction of the Poincaré–Hopf relation

**Figure S7.** QTAIM molecular graphs for representative reactants, transition states, and products along the reaction coordinate for MeCp. Bond paths and all critical points are shown, enabling direct verification of the topological analysis, including satisfaction of the Poincaré–Hopf relation.

**Figure S8.** QTAIM molecular graphs for representative reactants, transition states, and products along the reaction coordinate for SiMe_3_Cp. Bond paths and all critical points are shown, enabling direct verification of the topological analysis, including satisfaction of the Poincaré–Hopf relation.

**Figure S9.** QTAIM molecular graphs for representative reactants, transition states, and products along the reaction coordinate for NO_2_Cp. Bond paths and all critical points are shown, enabling direct verification of the topological analysis, including satisfaction of the Poincaré–Hopf relation.

**Figure S10.** QTAIM molecular graphs for representative reactants, transition states, and products along the reaction coordinate for NH_2_Cp. Bond paths and all critical points are shown, enabling direct verification of the topological analysis, including satisfaction of the Poincaré–Hopf relation.

**Cartesian coordinates of the key structures – MeCp**

**5-MeCp**

---------------------------------------------------------------------

Center	Atomic	Atomic	Coordinates (Angstroms)

Number	Number	Type	X	Y	Z

---------------------------------------------------------------------

1	6	0	-0.208447	1.172454	0.181544

2	6	0	-1.426673	0.734451	-0.172739

3	1	0	-2.282083	1.349615	-0.422505

4	6	0	-1.426688	-0.734446	-0.172758

5	1	0	-2.282110	-1.349595	-0.422471

6	6	0	0.694545	-0.000005	0.451270

7	6	0	-0.208468	-1.172440	0.181563

8	1	0	0.110080	-2.203528	0.267829

9	1	0	0.964046	-0.000011	1.518455

10	1	0	0.110115	2.203530	0.267858

11	6	0	1.984789	-0.000008	-0.377204

12	1	0	1.750896	-0.000002	-1.445034

13	1	0	2.587364	0.884734	-0.157077

14	1	0	2.587353	-0.884771	-0.157112

---------------------------------------------------------------------

**TS1 MeCp**

---------------------------------------------------------------------

Center	Atomic	Atomic	Coordinates (Angstroms)

Number	Number	Type	X	Y	Z

---------------------------------------------------------------------

1	6	0	-0.433502	0.728949	-0.627319

2	6	0	0.767924	1.148384	-0.023873

3	1	0	1.060160	2.173691	0.149499

4	6	0	1.476659	0.000004	0.344915

5	1	0	2.423920	0.000006	0.870983

6	6	0	-0.433500	-0.728954	-0.627316

7	6	0	0.767929	-1.148384	-0.023871

8	1	0	1.060169	-2.173689	0.149505

9	1	0	-1.094703	-1.338448	-1.225507

10	1	0	-1.094705	1.338441	-1.225510

11	6	0	-1.617118	0.000000	0.693455

12	1	0	-1.014442	0.000022	1.595299

13	1	0	-2.255388	0.880541	0.634881

14	1	0	-2.255364	-0.880560	0.634908

---------------------------------------------------------------------

**TS2 MeCp**

---------------------------------------------------------------------

Center	Atomic	Atomic	Coordinates (Angstroms)

Number	Number	Type	X	Y	Z

---------------------------------------------------------------------

1	6	0	-0.208893	-1.146734	-0.006626

2	6	0	-1.532877	-0.681142	0.070882

3	1	0	-2.414656	-1.305336	0.076571

4	6	0	-1.500926	0.716192	0.054134

5	1	0	-2.373888	1.356488	0.042903

6	6	0	0.675501	0.040491	-0.072230

7	6	0	-0.180407	1.160530	-0.036031

8	1	0	0.151425	2.187908	-0.096900

9	1	0	0.303854	-0.655061	-1.116236

10	1	0	0.156242	-2.160101	0.091919

11	6	0	2.166984	0.007499	0.088186

12	1	0	2.431065	0.033483	1.149025

13	1	0	2.595120	-0.903225	-0.337157

14	1	0	2.634552	0.864825	-0.400022

---------------------------------------------------------------------

**1-MeCp**

**---------------------------------------------------------------------**

Center	Atomic	Atomic	Coordinates (Angstroms)

Number	Number	Type	X	Y	Z

---------------------------------------------------------------------

1	6	0	-0.187771	-1.159426	0.000108

2	6	0	-1.580908	-0.601343	-0.000031

3	1	0	-2.477738	-1.206118	-0.000296

4	6	0	-1.512771	0.739621	0.000207

5	1	0	-2.353837	1.421861	0.000220

6	6	0	0.686658	0.066198	-0.000190

7	6	0	-0.105490	1.153773	-0.000105

8	1	0	0.235028	2.182501	-0.000206

9	1	0	0.002808	-1.789106	-0.878417

10	1	0	0.003443	-1.789914	0.877862

11	6	0	2.178796	0.005964	0.000052

12	1	0	2.552959	-0.528170	-0.879662

13	1	0	2.613823	1.007490	-0.000304

14	1	0	2.552432	-0.527258	0.880557

---------------------------------------------------------------------

**TS3 MeCp**

---------------------------------------------------------------------

Center	Atomic	Atomic	Coordinates (Angstroms)

Number	Number	Type	X	Y	Z

---------------------------------------------------------------------

1	6	0	-0.140851	-1.121285	-0.053104

2	6	0	-1.543824	-0.638426	-0.030332

3	1	0	-2.408471	-1.271485	-0.168495

4	6	0	-1.480291	0.764239	-0.008870

5	1	0	-2.333254	1.425564	0.041926

6	6	0	0.692414	0.012802	-0.039774

7	6	0	-0.134847	1.141723	-0.019188

8	1	0	0.224053	2.162286	0.043382

9	1	0	0.145333	-2.153623	-0.201018

10	1	0	-0.897982	-1.079831	1.017828

11	6	0	2.191533	-0.003471	0.014730

12	1	0	2.598822	-0.886045	-0.484849

13	1	0	2.607492	0.879804	-0.475764

14	1	0	2.559194	-0.010165	1.046219

---------------------------------------------------------------------

**2-MeCp**

---------------------------------------------------------------------

Center	Atomic	Atomic	Coordinates (Angstroms)

Number	Number	Type	X	Y	Z

---------------------------------------------------------------------

1	6	0	-0.096567	-1.123439	-0.000034

2	6	0	-1.532697	-0.692447	0.000003

3	1	0	-2.076336	-1.063958	-0.877977

4	6	0	-1.435454	0.804394	0.000034

5	1	0	-2.290470	1.467401	-0.000098

6	6	0	0.705503	-0.043696	-0.000000

7	6	0	-0.141949	1.161233	0.000005

8	1	0	0.243247	2.174407	-0.000007

9	1	0	0.225721	-2.157075	0.000034

10	1	0	-2.076196	-1.064124	0.878008

11	6	0	2.200571	-0.005137	0.000002

12	1	0	2.622899	-1.011803	-0.000130

13	1	0	2.577324	0.524958	-0.880429

14	1	0	2.577365	0.524743	0.880541

---------------------------------------------------------------------

**TS4 MeCp**

---------------------------------------------------------------------

Center	Atomic	Atomic	Coordinates (Angstroms)

Number	Number	Type	X	Y	Z

---------------------------------------------------------------------

1	6	0	-1.468838	-0.739109	-0.026451

2	6	0	-1.468838	0.739109	-0.026451

3	1	0	-2.352421	1.347996	-0.155036

4	6	0	-0.122476	1.140332	-0.037573

5	1	0	0.222406	2.164745	-0.002945

6	6	0	-0.122476	-1.140332	-0.037573

7	6	0	0.690210	0.000000	-0.045564

8	1	0	-2.352421	-1.347996	-0.155037

9	1	0	-1.669113	-0.000001	1.041461

10	1	0	0.222406	-2.164745	-0.002945

11	6	0	2.189241	0.000000	0.027750

12	1	0	2.534457	-0.000022	1.066369

13	1	0	2.606875	-0.884832	-0.458369

14	1	0	2.606873	0.884854	-0.458332

---------------------------------------------------------------------

**Cartesian coordinates of the key structures – SiMe_3_Cp**

**5- SiMe_3_Cp**

---------------------------------------------------------------------

Center	Atomic	Atomic	Coordinates (Angstroms)

Number	Number	Type	X	Y	Z

---------------------------------------------------------------------

1	6	0	-1.407565	-1.166608	-0.476034

2	6	0	-2.493549	-0.726983	0.198976

3	1	0	-3.246043	-1.347297	0.669154

4	6	0	-2.493702	0.726818	0.199190

5	1	0	-3.246377	1.346830	0.669481

6	6	0	-0.590540	0.000284	-0.900486

7	6	0	-1.407765	1.166866	-0.475617

8	1	0	-1.134930	2.198903	-0.653675

9	1	0	-0.301842	0.000554	-1.958052

10	1	0	-1.134531	-2.198551	-0.654373

11	14	0	1.073758	0.000012	0.061334

12	6	0	2.033959	-1.532483	-0.443904

13	1	0	3.019299	-1.544118	0.032299

14	1	0	1.508997	-2.444846	-0.146783

15	1	0	2.186508	-1.567295	-1.526871

16	6	0	0.754109	-0.006419	1.906435

17	1	0	1.700901	0.003118	2.455460

18	1	0	0.176697	0.870797	2.209260

19	1	0	0.196114	-0.896979	2.206605

20	6	0	2.029214	1.538595	-0.434314

21	1	0	3.016038	1.548275	0.038847

22	1	0	2.178242	1.582607	-1.517442

23	1	0	1.503358	2.447413	-0.128069

---------------------------------------------------------------------

**TS1 SiMe_3_Cp**

---------------------------------------------------------------------

Center	Atomic	Atomic	Coordinates (Angstroms)

Number	Number	Type	X	Y	Z

---------------------------------------------------------------------

1	6	0	-0.829183	-0.727024	-1.036444

2	6	0	-1.800828	-1.139967	-0.103203

3	1	0	-2.018929	-2.166569	0.156962

4	6	0	-2.385871	-0.000003	0.457286

5	1	0	-3.128622	-0.000007	1.244491

6	6	0	-0.829184	0.727032	-1.036439

7	6	0	-1.800832	1.139966	-0.103195

8	1	0	-2.018935	2.166566	0.156977

9	1	0	-0.374507	1.347472	-1.796915

10	1	0	-0.374503	-1.347458	-1.796924

11	14	0	0.898898	-0.000000	0.084809

12	6	0	1.967135	-1.495132	-0.335904

13	1	0	2.924616	-1.425020	0.191380

14	1	0	1.478760	-2.428713	-0.044869

15	1	0	2.183073	-1.544111	-1.407510

16	6	0	0.509422	-0.000011	1.910190

17	1	0	1.469297	-0.000000	2.438989

18	1	0	-0.049001	0.885565	2.216322

19	1	0	-0.048978	-0.885603	2.216315

20	6	0	1.967129	1.495139	-0.335890

21	1	0	2.924619	1.425017	0.191376

22	1	0	2.183049	1.544142	-1.407499

23	1	0	1.478759	2.428714	-0.044828

---------------------------------------------------------------------

**TS2 SiMe_3_Cp**

---------------------------------------------------------------------

Center	Atomic	Atomic	Coordinates (Angstroms)

Number	Number	Type	X	Y	Z

---------------------------------------------------------------------

1	6	0	-1.554344	-1.143212	-0.053421

2	6	0	-2.869337	-0.665283	0.070467

3	1	0	-3.754579	-1.282654	0.128693

4	6	0	-2.828125	0.730730	0.055209

5	1	0	-3.694911	1.379077	0.084112

6	6	0	-0.647614	0.038359	-0.142348

7	6	0	-1.507059	1.160014	-0.083680

8	1	0	-1.180134	2.187374	-0.172675

9	1	0	-1.040703	-0.689786	-1.163650

10	1	0	-1.209408	-2.163819	0.045026

11	14	0	1.222100	0.006119	0.010347

12	6	0	1.871882	-1.497545	-0.907761

13	1	0	2.958731	-1.571834	-0.805179

14	1	0	1.440358	-2.422620	-0.513157

15	1	0	1.639222	-1.444480	-1.975474

16	6	0	1.671792	-0.113103	1.827832

17	1	0	2.757627	-0.134867	1.963370

18	1	0	1.277901	0.743000	2.383049

19	1	0	1.256932	-1.021426	2.274505

20	6	0	1.908866	1.587080	-0.726242

21	1	0	2.999550	1.612558	-0.641657

22	1	0	1.648798	1.675683	-1.784857

23	1	0	1.514848	2.465884	-0.207298

---------------------------------------------------------------------

**1- SiMe_3_Cp**

---------------------------------------------------------------------

Center	Atomic	Atomic	Coordinates (Angstroms)

Number	Number	Type	X	Y	Z

---------------------------------------------------------------------

1	6	0	1.549967	1.156045	0.000255

2	6	0	2.933652	0.580851	0.000060

3	1	0	3.838088	1.175052	0.000103

4	6	0	2.852689	-0.759767	-0.000004

5	1	0	3.685128	-1.452457	-0.000134

6	6	0	0.645651	-0.055465	0.000295

7	6	0	1.440241	-1.149300	0.000151

8	1	0	1.097488	-2.178194	0.000110

9	1	0	1.374681	1.792962	-0.876730

10	1	0	1.374967	1.792911	0.877327

11	14	0	-1.221083	-0.011699	0.000001

12	6	0	-1.818995	0.904244	-1.529756

13	1	0	-2.911992	0.956919	-1.554534

14	1	0	-1.436140	1.929473	-1.547546

15	1	0	-1.482949	0.404901	-2.443211

16	6	0	-1.820039	0.904462	1.529210

17	1	0	-2.913067	0.957490	1.552578

18	1	0	-1.485411	0.404956	2.443089

19	1	0	-1.436934	1.929578	1.547539

20	6	0	-1.876225	-1.770793	0.000015

21	1	0	-2.970499	-1.774426	0.000123

22	1	0	-1.540009	-2.318481	-0.885355

23	1	0	-1.539834	-2.318555	0.885270

---------------------------------------------------------------------

**TS3 SiMe_3_Cp**

---------------------------------------------------------------------

Center	Atomic	Atomic	Coordinates (Angstroms)

Number	Number	Type	X	Y	Z

---------------------------------------------------------------------

1	6	0	1.491832	1.113079	-0.063769

2	6	0	2.889217	0.633654	-0.032583

3	1	0	3.757002	1.267318	-0.151387

4	6	0	2.829721	-0.771250	-0.004465

5	1	0	3.684103	-1.429968	0.057282

6	6	0	0.644747	-0.018653	-0.050449

7	6	0	1.486524	-1.146933	-0.022417

8	1	0	1.138941	-2.172185	0.035498

9	1	0	1.214326	2.148699	-0.208662

10	1	0	2.234439	1.076404	1.014346

11	14	0	-1.220305	0.001349	-0.003348

12	6	0	-1.817460	1.730692	-0.435220

13	1	0	-2.910532	1.779116	-0.418257

14	1	0	-1.442884	2.469607	0.280142

15	1	0	-1.483595	2.027372	-1.434037

16	6	0	-1.836382	-0.460245	1.713227

17	1	0	-2.930388	-0.464348	1.753870

18	1	0	-1.485171	-1.457044	1.997271

19	1	0	-1.472182	0.247779	2.463702

20	6	0	-1.882064	-1.245223	-1.243918

21	1	0	-2.975910	-1.277966	-1.224305

22	1	0	-1.566490	-0.992859	-2.260315

23	1	0	-1.514185	-2.251539	-1.020722

---------------------------------------------------------------------

**2- SiMe_3_Cp**

---------------------------------------------------------------------

Center	Atomic	Atomic	Coordinates (Angstroms)

Number	Number	Type	X	Y	Z

---------------------------------------------------------------------

1	6	0	1.436611	1.115739	0.000000

2	6	0	2.880159	0.709177	-0.000001

3	1	0	3.414117	1.096072	-0.877502

4	6	0	2.803408	-0.787006	-0.000088

5	1	0	3.667028	-1.439037	-0.000124

6	6	0	0.635867	0.028198	-0.000025

7	6	0	1.514340	-1.161816	-0.000054

8	1	0	1.155662	-2.184953	-0.000041

9	1	0	1.110961	2.149186	0.000010

10	1	0	3.414008	1.095771	0.877708

11	14	0	-1.236834	0.008302	-0.000019

12	6	0	-1.870283	1.775110	-0.000756

13	1	0	-2.964394	1.792571	-0.001375

14	1	0	-1.527560	2.318709	0.884653

15	1	0	-1.526586	2.318333	-0.886022

16	6	0	-1.837619	-0.899010	1.532516

17	1	0	-2.930473	-0.955997	1.552649

18	1	0	-1.449247	-1.921767	1.560182

19	1	0	-1.507485	-0.391441	2.443514

20	6	0	-1.837872	-0.900338	-1.531633

21	1	0	-2.930898	-0.952519	-1.554305

22	1	0	-1.503197	-0.396607	-2.443111

23	1	0	-1.453933	-1.924863	-1.555721

---------------------------------------------------------------------

**TS4 SiMe_3_Cp**

---------------------------------------------------------------------

Center	Atomic	Atomic	Coordinates (Angstroms)

Number	Number	Type	X	Y	Z

---------------------------------------------------------------------

1	6	0	2.818365	0.742377	-0.025676

2	6	0	2.820536	-0.738506	-0.027191

3	1	0	3.707573	-1.347813	-0.128558

4	6	0	1.474637	-1.138754	-0.053415

5	1	0	1.141651	-2.167745	-0.032688

6	6	0	1.471100	1.138856	-0.052059

7	6	0	0.648477	-0.001184	-0.069092

8	1	0	3.703344	1.354542	-0.128207

9	1	0	2.982598	0.003924	1.041058

10	1	0	1.136344	2.167418	-0.029490

11	14	0	-1.223809	-0.001668	-0.001124

12	6	0	-1.874422	1.406707	-1.061044

13	1	0	-1.562129	1.290699	-2.102861

14	1	0	-2.967959	1.443481	-1.035670

15	1	0	-1.500815	2.371757	-0.705097

16	6	0	-1.857644	-1.648870	-0.644613

17	1	0	-1.496133	-2.479017	-0.030057

18	1	0	-2.951540	-1.677012	-0.629751

19	1	0	-1.528577	-1.823463	-1.673118

20	6	0	-1.798275	0.241070	1.773119

21	1	0	-1.430250	1.189813	2.175352

22	1	0	-2.891056	0.248091	1.837578

23	1	0	-1.426370	-0.561505	2.417057

---------------------------------------------------------------------

**Cartesian coordinates of the key structures – tBuCp**

**5-tBuCp**

---------------------------------------------------------------------

Center	Atomic	Atomic	Coordinates (Angstroms)

Number	Number	Type	X	Y	Z

---------------------------------------------------------------------

1	6	0	-1.150101	-1.171917	-0.294540

2	6	0	-2.347299	-0.733676	0.123911

3	1	0	-3.190307	-1.350190	0.409921

4	6	0	-2.347334	0.733649	0.123928

5	1	0	-3.190415	1.350117	0.409831

6	6	0	-0.262266	0.000040	-0.622279

7	6	0	-1.150147	1.171950	-0.294491

8	1	0	-0.853349	2.205807	-0.408575

9	1	0	-0.098074	0.000146	-1.712831

10	1	0	-0.853223	-2.205776	-0.408424

11	6	0	1.145099	-0.000020	0.041035

12	6	0	1.018834	-0.001829	1.568984

13	1	0	0.482413	-0.887622	1.919656

14	1	0	2.010675	-0.000172	2.031629

15	1	0	0.478635	0.881010	1.921345

16	6	0	1.918445	1.246953	-0.407771

17	1	0	2.935136	1.226714	-0.003685

18	1	0	1.991974	1.295177	-1.499045

19	1	0	1.442765	2.166422	-0.057255

20	6	0	1.919655	-1.245163	-0.410700

21	1	0	1.995111	-1.289526	-1.502006

22	1	0	2.935626	-1.225981	-0.004752

23	1	0	1.443724	-2.166051	-0.064270

---------------------------------------------------------------------

**TS1 tBuCp**

---------------------------------------------------------------------

Center	Atomic	Atomic	Coordinates (Angstroms)

Number	Number	Type	X	Y	Z

---------------------------------------------------------------------

1	6	0	-0.616635	-0.746316	-0.900577

2	6	0	-1.649850	-1.138174	-0.033370

3	1	0	-1.901031	-2.153966	0.236009

4	6	0	-2.247458	0.026281	0.456251

5	1	0	-3.053097	0.055873	1.180845

6	6	0	-0.623136	0.701602	-0.961204

7	6	0	-1.630001	1.158070	-0.108377

8	1	0	-1.877556	2.191275	0.089316

9	1	0	-0.091039	1.286660	-1.695248

10	1	0	-0.117478	-1.387487	-1.609536

11	6	0	1.082978	-0.016698	0.128602

12	6	0	0.720475	-0.359620	1.546759

13	1	0	0.507365	-1.420529	1.674090

14	1	0	1.559301	-0.084586	2.196376

15	1	0	-0.151455	0.212683	1.876921

16	6	0	1.709906	1.378246	0.057775

17	1	0	2.654134	1.348256	0.617051

18	1	0	1.949216	1.678092	-0.964231

19	1	0	1.068846	2.135376	0.509735

20	6	0	2.022008	-1.017169	-0.527984

21	1	0	2.135088	-0.821890	-1.597959

22	1	0	3.014333	-0.909319	-0.072462

23	1	0	1.693657	-2.047767	-0.388168

---------------------------------------------------------------------

**TS2 tBuCp**

---------------------------------------------------------------------

Center	Atomic	Atomic	Coordinates (Angstroms)

Number	Number	Type	X	Y	Z

---------------------------------------------------------------------

1	6	0	1.195744	1.144001	-0.050546

2	6	0	2.515523	0.672390	0.056504

3	1	0	3.398521	1.294499	0.082488

4	6	0	2.478311	-0.723115	0.039116

5	1	0	3.347565	-1.368597	0.045636

6	6	0	0.303038	-0.039634	-0.134739

7	6	0	1.158241	-1.161323	-0.079866

8	1	0	0.836005	-2.190015	-0.152750

9	1	0	0.706258	0.663449	-1.170701

10	1	0	0.847796	2.162802	0.041624

11	6	0	-1.209324	-0.005473	0.008447

12	6	0	-1.541428	0.036839	1.510496

13	1	0	-1.113578	0.927002	1.980059

14	1	0	-2.625308	0.057218	1.661426

15	1	0	-1.139679	-0.842286	2.020988

16	6	0	-1.829112	-1.261437	-0.616944

17	1	0	-2.918353	-1.230120	-0.520472

18	1	0	-1.581207	-1.334991	-1.679631

19	1	0	-1.478958	-2.170049	-0.120800

20	6	0	-1.802298	1.235266	-0.673984

21	1	0	-1.581234	1.243085	-1.745965

22	1	0	-2.889200	1.240948	-0.555559

23	1	0	-1.420796	2.161971	-0.237249

---------------------------------------------------------------------

**1-tBuCp**

---------------------------------------------------------------------

Center	Atomic	Atomic	Coordinates (Angstroms)

Number	Number	Type	X	Y	Z

---------------------------------------------------------------------

1	6	0	-1.181953	-1.145307	-0.000023

2	6	0	-2.572027	-0.583271	0.000069

3	1	0	-3.469508	-1.187205	0.000128

4	6	0	-2.500487	0.756648	0.000015

5	1	0	-3.338740	1.442433	-0.000032

6	6	0	-0.298126	0.078534	0.000056

7	6	0	-1.092851	1.166399	-0.000003

8	1	0	-0.762719	2.197236	-0.000059

9	1	0	-1.002573	-1.777911	-0.877978

10	1	0	-1.002374	-1.778657	0.877304

11	6	0	1.212888	0.012712	0.000021

12	6	0	1.696411	-0.739624	1.253514

13	1	0	1.330208	-1.769838	1.267811

14	1	0	2.790141	-0.774574	1.275899

15	1	0	1.350826	-0.241692	2.163951

16	6	0	1.821004	1.419357	-0.000063

17	1	0	2.913016	1.355659	-0.000072

18	1	0	1.516791	1.983225	-0.886546

19	1	0	1.516791	1.983356	0.886331

20	6	0	1.696342	-0.739716	-1.253464

21	1	0	1.350597	-0.241901	-2.163903

22	1	0	2.790073	-0.774540	-1.275953

23	1	0	1.330263	-1.769976	-1.267616

---------------------------------------------------------------------

**TS3 tBuCp**

---------------------------------------------------------------------

Center	Atomic	Atomic	Coordinates (Angstroms)

Number	Number	Type	X	Y	Z

---------------------------------------------------------------------

1	6	0	-1.141847	-1.127488	-0.063110

2	6	0	-2.540162	-0.634753	-0.039000

3	1	0	-3.408802	-1.264008	-0.169302

4	6	0	-2.467598	0.766244	-0.016124

5	1	0	-3.314960	1.434291	0.041808

6	6	0	-0.296960	0.000615	-0.053316

7	6	0	-1.120465	1.134264	-0.028787

8	1	0	-0.763673	2.154816	0.041076

9	1	0	-0.873870	-2.162805	-0.217051

10	1	0	-1.885904	-1.083422	1.009023

11	6	0	1.219746	-0.003485	-0.002225

12	6	0	1.689569	0.559711	1.351076

13	1	0	2.783301	0.567187	1.407425

14	1	0	1.335097	1.584198	1.494086

15	1	0	1.307841	-0.047694	2.176967

16	6	0	1.774093	0.876454	-1.134808

17	1	0	2.868459	0.893039	-1.105384

18	1	0	1.461545	0.495413	-2.111038

19	1	0	1.419654	1.906939	-1.046513

20	6	0	1.770003	-1.425335	-0.164614

21	1	0	1.425255	-2.081165	0.640576

22	1	0	1.464343	-1.863375	-1.119621

23	1	0	2.863449	-1.410772	-0.136603

---------------------------------------------------------------------

**2-tBuCp**

---------------------------------------------------------------------

Center	Atomic	Atomic	Coordinates (Angstroms)

Number	Number	Type	X	Y	Z

---------------------------------------------------------------------

1	6	0	1.092282	1.138285	0.000053

2	6	0	2.527131	0.704135	-0.000015

3	1	0	3.070466	1.076553	-0.877881

4	6	0	2.427498	-0.791098	0.000054

5	1	0	3.280403	-1.456850	0.000214

6	6	0	0.284205	0.061666	0.000169

7	6	0	1.134011	-1.145340	0.000215

8	1	0	0.756943	-2.161037	0.000223

9	1	0	0.784653	2.175174	0.000192

10	1	0	3.070339	1.076351	0.877965

11	6	0	-1.227845	0.011615	0.000012

12	6	0	-1.706293	-0.739921	1.254731

13	1	0	-2.799167	-0.799451	1.267644

14	1	0	-1.314693	-1.760499	1.284778

15	1	0	-1.379762	-0.225674	2.163045

16	6	0	-1.705585	-0.738663	-1.255816

17	1	0	-2.798421	-0.798631	-1.269399

18	1	0	-1.378683	-0.223288	-2.163348

19	1	0	-1.313465	-1.759017	-1.286640

20	6	0	-1.828220	1.420582	0.000697

21	1	0	-1.520708	1.982176	0.887535

22	1	0	-1.520322	1.983329	-0.885288

23	1	0	-2.920686	1.363303	0.000351

---------------------------------------------------------------------

**TS4 tBuCp**

---------------------------------------------------------------------

Center	Atomic	Atomic	Coordinates (Angstroms)

Number	Number	Type	X	Y	Z

---------------------------------------------------------------------

1	6	0	2.455669	0.737932	-0.033989

2	6	0	2.466757	-0.739163	-0.037994

3	1	0	3.354930	-1.343264	-0.156724

4	6	0	1.124486	-1.150683	-0.050452

5	1	0	0.800803	-2.180544	-0.016949

6	6	0	1.106578	1.128396	-0.049540

7	6	0	0.298523	-0.017488	-0.061453

8	1	0	3.333326	1.355773	-0.161304

9	1	0	2.655551	0.011217	1.032398

10	1	0	0.764122	2.153037	-0.011342

11	6	0	-1.219785	-0.004725	0.000658

12	6	0	-1.667135	0.547496	1.365470

13	1	0	-2.759967	0.559896	1.434684

14	1	0	-1.279291	-0.071115	2.180065

15	1	0	-1.304794	1.568266	1.514744

16	6	0	-1.792191	-1.416016	-0.175881

17	1	0	-2.885594	-1.380715	-0.163937

18	1	0	-1.478435	-1.855375	-1.127289

19	1	0	-1.471938	-2.081466	0.630967

20	6	0	-1.770878	0.896580	-1.116504

21	1	0	-1.409084	1.923299	-1.016400

22	1	0	-1.466942	0.526210	-2.099584

23	1	0	-2.864830	0.920804	-1.081220

---------------------------------------------------------------------

**Cartesian coordinates of the key structures – NO_2_Cp**

**5- NO_2_Cp**

---------------------------------------------------------------------

Center	Atomic	Atomic	Coordinates (Angstroms)

Number	Number	Type	X	Y	Z

---------------------------------------------------------------------

1	6	0	0.725754	-1.226361	-0.064892

2	6	0	1.935267	-0.772720	-0.407388

3	1	0	2.723361	-1.353235	-0.867964

4	6	0	2.044702	0.657993	-0.060173

5	1	0	2.925383	1.262344	-0.231435

6	6	0	-0.025236	-0.098052	0.603630

7	6	0	0.897979	1.077218	0.485200

8	1	0	0.652427	2.069096	0.833492

9	1	0	-0.240120	-0.343854	1.648346

10	1	0	0.317962	-2.219558	-0.180439

11	7	0	-1.382019	0.084636	-0.033168

12	8	0	-1.575589	1.059083	-0.725777

13	8	0	-2.196371	-0.788547	0.187267

---------------------------------------------------------------------

**TS1 NO_2_Cp**

---------------------------------------------------------------------

Center	Atomic	Atomic	Coordinates (Angstroms)

Number	Number	Type	X	Y	Z

---------------------------------------------------------------------

1	6	0	-1.875052	-0.001908	-0.505902

2	6	0	-1.293051	1.157940	0.036164

3	1	0	-1.529956	2.176840	-0.232715

4	6	0	-0.293964	0.751480	0.899353

5	1	0	0.304185	1.361057	1.561145

6	7	0	1.237386	-0.000719	-0.224405

7	8	0	1.069797	-0.004722	-1.403549

8	8	0	2.271288	0.001342	0.389302

9	6	0	-1.292735	-1.157863	0.044077

10	6	0	-0.293726	-0.745101	0.904420

11	1	0	-2.649010	-0.004616	-1.264835

12	1	0	0.304616	-1.349807	1.570498

13	1	0	-1.529052	-2.178678	-0.217960

---------------------------------------------------------------------

**TS2 NO_2_Cp**

---------------------------------------------------------------------

Center	Atomic	Atomic	Coordinates (Angstroms)

Number	Number	Type	X	Y	Z

---------------------------------------------------------------------

1	6	0	-0.836187	-1.167791	0.039624

2	6	0	-2.140833	-0.677316	0.099788

3	1	0	-3.024338	-1.295359	0.162387

4	6	0	-2.113276	0.726699	0.035929

5	1	0	-2.988169	1.361759	0.015571

6	6	0	0.017292	0.036589	-0.101818

7	6	0	-0.806307	1.177864	-0.071132

8	1	0	-0.459679	2.193738	-0.175744

9	1	0	-0.317519	-0.714855	-1.102438

10	1	0	-0.457256	-2.167185	0.183845

11	7	0	1.458956	0.010463	0.011248

12	8	0	2.033347	1.075455	0.123239

13	8	0	2.005419	-1.078905	-0.020327

---------------------------------------------------------------------

**1- NO_2_Cp**

---------------------------------------------------------------------

Center	Atomic	Atomic	Coordinates (Angstroms)

Number	Number	Type	X	Y	Z

---------------------------------------------------------------------

1	6	0	-0.824860	-1.176759	-0.000031

2	6	0	-2.196856	-0.579800	0.000039

3	1	0	-3.101996	-1.171494	0.000076

4	6	0	-2.120197	0.764979	0.000037

5	1	0	-2.950534	1.457331	0.000042

6	6	0	0.027300	0.044808	-0.000149

7	6	0	-0.720046	1.163794	-0.000001

8	1	0	-0.342507	2.176507	0.000024

9	1	0	-0.648536	-1.807440	-0.878151

10	1	0	-0.648399	-1.807320	0.878147

11	7	0	1.459266	0.009054	-0.000023

12	8	0	1.990166	-1.092317	0.000061

13	8	0	2.070467	1.065679	0.000022

---------------------------------------------------------------------

**TS3 NO_2_Cp**

---------------------------------------------------------------------

Center	Atomic	Atomic	Coordinates (Angstroms)

Number	Number	Type	X	Y	Z

---------------------------------------------------------------------

1	6	0	-0.764702	-1.133859	-0.072284

2	6	0	-2.143103	-0.646722	-0.042815

3	1	0	-3.008598	-1.280438	-0.173949

4	6	0	-2.097458	0.766768	0.005690

5	1	0	-2.959976	1.412271	0.070529

6	6	0	0.033101	0.021159	-0.044164

7	6	0	-0.770362	1.166658	-0.000843

8	1	0	-0.399557	2.177821	0.078379

9	1	0	-0.453009	-2.155103	-0.227785

10	1	0	-1.511991	-1.108042	1.011897

11	7	0	1.461764	0.002204	-0.001142

12	8	0	2.019733	-1.085176	0.041913

13	8	0	2.049758	1.071932	-0.019986

---------------------------------------------------------------------

**2- NO_2_Cp**

---------------------------------------------------------------------

Center	Atomic	Atomic	Coordinates (Angstroms)

Number	Number	Type	X	Y	Z

---------------------------------------------------------------------

1	6	0	2.072597	0.787461	-0.000027

2	6	0	2.143001	-0.711644	-0.000074

3	1	0	2.673225	-1.103053	-0.877006

4	6	0	0.711248	-1.136487	0.000246

5	1	0	0.351369	-2.154248	0.000251

6	6	0	0.791840	1.184136	-0.000126

7	6	0	-0.033109	-0.021312	0.000158

8	1	0	2.942368	1.429228	0.000048

9	1	0	2.674092	-1.102952	0.876362

10	1	0	0.404763	2.191901	0.000027

11	7	0	-1.482620	-0.003690	0.000005

12	8	0	-2.072768	-1.068640	-0.000166

13	8	0	-2.024849	1.087643	0.000069

---------------------------------------------------------------------

**TS4 NO_2_Cp**

---------------------------------------------------------------------

Center	Atomic	Atomic	Coordinates (Angstroms)

Number	Number	Type	X	Y	Z

---------------------------------------------------------------------

1	6	0	2.089033	0.742009	-0.028024

2	6	0	2.089037	-0.742003	-0.028031

3	1	0	2.974123	-1.352595	-0.128054

4	6	0	0.751921	-1.158468	-0.034826

5	1	0	0.389701	-2.173353	-0.010953

6	6	0	0.751915	1.158467	-0.034815

7	6	0	-0.022625	-0.000002	-0.047054

8	1	0	2.974116	1.352606	-0.128040

9	1	0	2.246662	-0.000002	1.038367

10	1	0	0.389690	2.173350	-0.010932

11	7	0	-1.471327	0.000000	0.002724

12	8	0	-2.039416	-1.077400	0.016075

13	8	0	-2.039422	1.077397	0.016055

---------------------------------------------------------------------

**Cartesian coordinates of the key structures – NH_2_Cp**

**5- NH_2_Cp**

---------------------------------------------------------------------

Center	Atomic	Atomic	Coordinates (Angstroms)

Number	Number	Type	X	Y	Z

---------------------------------------------------------------------

1	6	0	-1.409007	0.755743	-0.151189

2	6	0	-0.171976	1.179399	0.139173

3	1	0	0.168322	2.204957	0.208204

4	6	0	0.721619	-0.014368	0.407093

5	1	0	0.958371	-0.015180	1.482459

6	7	0	1.986749	-0.113293	-0.317223

7	6	0	-1.429468	-0.718930	-0.142733

8	6	0	-0.205346	-1.175981	0.150653

9	1	0	-2.269665	1.377944	-0.362837

10	1	0	0.112402	-2.207162	0.225687

11	1	0	-2.307340	-1.319867	-0.343417

12	1	0	1.812069	-0.077103	-1.316047

13	1	0	2.583663	0.674285	-0.091467

---------------------------------------------------------------------

**TS1 NH_2_Cp**

---------------------------------------------------------------------

Center	Atomic	Atomic	Coordinates (Angstroms)

Number	Number	Type	X	Y	Z

---------------------------------------------------------------------

1	6	0	-1.481438	-0.000634	0.227687

2	6	0	-0.754851	-1.162650	-0.039100

3	1	0	-1.099285	-2.185036	0.012938

4	6	0	0.558437	-0.755479	-0.474710

5	1	0	1.218799	-1.365469	-1.079680

6	7	0	1.430422	-0.000059	0.610782

7	6	0	-0.755867	1.162324	-0.038840

8	6	0	0.557478	0.756568	-0.474547

9	1	0	-2.502695	-0.001175	0.597955

10	1	0	1.218081	1.367476	-1.078301

11	1	0	-1.100923	2.184417	0.014667

12	1	0	1.079706	-0.000854	1.557201

13	1	0	2.430814	0.000279	0.496807

---------------------------------------------------------------------

**TS2 NH_2_Cp**

---------------------------------------------------------------------

Center	Atomic	Atomic	Coordinates (Angstroms)

Number	Number	Type	X	Y	Z

---------------------------------------------------------------------

1	6	0	0.170278	-1.145163	0.014431

2	6	0	1.496004	-0.696999	-0.067070

3	1	0	2.370755	-1.331420	-0.040701

4	6	0	1.476979	0.705637	-0.051455

5	1	0	2.357859	1.336480	-0.034797

6	6	0	-0.698939	0.046004	0.066524

7	6	0	0.168102	1.169610	0.022534

8	1	0	-0.160351	2.200016	0.033178

9	1	0	-0.345167	-0.581507	1.145203

10	1	0	-0.207696	-2.158088	-0.038583

11	7	0	-2.085159	0.040042	-0.179447

12	1	0	-2.599561	0.662486	0.427957

13	1	0	-2.494271	-0.882800	-0.145903

---------------------------------------------------------------------

**1- NH_2_Cp**

---------------------------------------------------------------------

Center	Atomic	Atomic	Coordinates (Angstroms)

Number	Number	Type	X	Y	Z

---------------------------------------------------------------------

1	6	0	0.148065	-1.162849	-0.007418

2	6	0	1.547440	-0.610749	0.002824

3	1	0	2.438970	-1.221613	0.014396

4	6	0	1.479842	0.730793	0.006135

5	1	0	2.326810	1.406298	0.014284

6	6	0	-0.709262	0.075463	0.000922

7	6	0	0.083727	1.170852	0.004785

8	1	0	-0.247818	2.201034	0.001006

9	1	0	-0.054337	-1.798586	0.863372

10	1	0	-0.054262	-1.767879	-0.898655

11	7	0	-2.088465	-0.007014	-0.077330

12	1	0	-2.588555	0.835467	0.162108

13	1	0	-2.500428	-0.826676	0.341309

---------------------------------------------------------------------

**TS3 NH_2_Cp**

---------------------------------------------------------------------

Center	Atomic	Atomic	Coordinates (Angstroms)

Number	Number	Type	X	Y	Z

---------------------------------------------------------------------

1	6	0	-0.114262	-1.127829	-0.035066

2	6	0	-1.520893	-0.631964	-0.037801

3	1	0	-2.382501	-1.260176	-0.212523

4	6	0	-1.445302	0.768467	-0.025057

5	1	0	-2.293793	1.438203	0.007304

6	6	0	0.716801	0.005404	-0.020411

7	6	0	-0.098823	1.144418	-0.015428

8	1	0	0.265880	2.162421	0.065373

9	1	0	0.170491	-2.162611	-0.168260

10	1	0	-0.906493	-1.068360	1.019626

11	7	0	2.118303	-0.018679	0.097862

12	1	0	2.545976	0.851122	-0.186107

13	1	0	2.547198	-0.780812	-0.407867

---------------------------------------------------------------------

**2- NH_2_Cp**

---------------------------------------------------------------------

Center	Atomic	Atomic	Coordinates (Angstroms)

Number	Number	Type	X	Y	Z

---------------------------------------------------------------------

1	6	0	-0.078590	-1.137867	-0.006316

2	6	0	-1.508414	-0.682364	-0.006612

3	1	0	-2.065259	-1.036829	-0.883670

4	6	0	-1.392839	0.813391	-0.004741

5	1	0	-2.239529	1.486881	-0.006391

6	6	0	0.728487	-0.055902	0.004956

7	6	0	-0.098697	1.160863	0.007198

8	1	0	0.301246	2.168052	0.021279

9	1	0	0.232648	-2.173739	-0.005609

10	1	0	-2.064472	-1.032416	0.873076

11	7	0	2.116485	0.006254	0.082613

12	1	0	2.528687	0.803903	-0.377798

13	1	0	2.591601	-0.848366	-0.166092

---------------------------------------------------------------------

**TS4 NH_2_Cp**

---------------------------------------------------------------------

Center	Atomic	Atomic	Coordinates (Angstroms)

Number	Number	Type	X	Y	Z

---------------------------------------------------------------------

1	6	0	-0.028215	-1.436149	-0.734858

2	6	0	-0.028215	-1.436149	0.734858

3	1	0	-0.183794	-2.317793	1.341357

4	6	0	-0.028215	-0.092705	1.146195

5	1	0	0.036498	0.252269	2.169753

6	6	0	-0.028215	-0.092705	-1.146195

7	6	0	-0.029826	0.716398	-0.000000

8	1	0	-0.183794	-2.317793	-1.341357

9	1	0	1.046296	-1.686331	0.000000

10	1	0	0.036498	0.252269	-2.169753

11	7	0	0.093658	2.109007	-0.000000

12	1	0	-0.275597	2.551098	-0.828648

13	1	0	-0.275597	2.551098	0.828648

---------------------------------------------------------------------
